# Assessing the Transferability of Species Distribution Models: A Cross‐Continental Evaluation

**DOI:** 10.1002/ece3.73534

**Published:** 2026-04-29

**Authors:** Takayuki Matsui

**Affiliations:** ^1^ Graduate School of Agriculture, Kyoto University Kyoto Japan

**Keywords:** AUC, Boyce index, ecological niche model, Maxent, species distribution model, transferability

## Abstract

Model transferability is essential for predicting species distribution in novel regions or time periods. However, assessing model transferability remains a major knowledge gap. The aim of this study was to identify an appropriate method for assessing the transferability of species distribution models (SDMs) in validation, specifically focusing on the types of test datasets and evaluators. The model evaluation ability of validation methods was examined using several completely independent datasets that are rarely used in other studies. The distribution of three invasive plant species was predicted using Maxent across different continents with datasets sourced from various continental combinations. The evaluation ability for testing on internal random holdout datasets was examined by comparing the evaluator values from internal cross‐validations and aforementioned predictions. Subsequently, the paired distribution predictions from identical models in different regions were compared to examine the evaluation ability for testing on spatially independent datasets. The relationship between the paired predictions mirrored that between the predictions using the test datasets and predictions in the target regions. Three evaluators were compared: the area under the receiver operating characteristic curve (AUC), continuous Boyce index (CBI), and correlation coefficient with the predictions from the model calibrated in the predicted region (RWIP). Cross‐validation using random holdout datasets consistently rated all models as good; however, the prediction evaluations in the target regions varied widely. Therefore, conventional cross‐validation proved inadequate for assessing model transferability. Analyzing the paired distribution predictions from the same models across different regions revealed that using the AUC and CBI increased the evaluation uncertainty, whereas applying the RWIP maintained relatively low evaluation uncertainty. This study confirms that the conventional approach, namely using random holdout test datasets and the AUC, fails to reliably assess model transferability. Instead, using spatially independent test datasets and the RWIP provides a more robust evaluation approach.

## Graphical abstract

This study confirms that the conventional approach, namely using random holdout test datasets and the AUC, fails to reliably assess model transferability. Instead, using spatially independent test datasets and the RWIP provides a more robust evaluation approach. Cross‐validation using random holdout datasets failed to distinguish whether the model had good or poor predictive performance in the target region. In the comparison between paired distribution predictions from the same models in different regions, a strong correlation was observed exclusively when the RWIP was used.

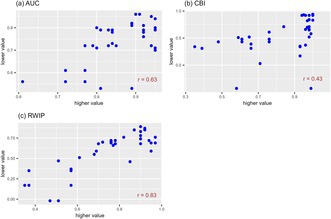

## 1 Introduction

Species distribution models (SDMs), also referred to as ecological niche models, are increasingly utilized to assess the invasion potential of species in novel regions and predict the impacts of climate change on species distributions (Araújo et al. 2019: fig. 1). These studies have been executed under the assumption that the models can reliably predict species responses in new scenarios, an aspect known as model transferability.

Yates et al. (2018) identified priority knowledge gaps that, if addressed, could substantially improve ecological model transfers. Among these, the question of assessing transferability emerged as the most pressing knowledge gap in discussions involving 50 experts. Transferability can be assessed by comparing the distribution prediction results with distribution data from the target system. (Yates et al. (2018) defines “target system” as “the system to which a model is transferred”.) However, when SDMs are utilized to predict the species distribution in a new geographical region or a different time period, the species distribution in the target system is usually unknown. Consequently, in model validation, transferability is assessed using a test dataset containing distribution data. The priority knowledge gap is “How should transferability be assessed in model validation?” (Yates et al. 2018).

Fourcade et al. (2018) highlighted flaws in current SDM validation practices, showing that models constructed using arbitrary variables (such as those derived from paintings) as input environmental predictors are often classified as good according to the most widely used evaluation methods. Their findings underscored the need for a critical assessment of SDM validation practices.

**FIGURE 1:**
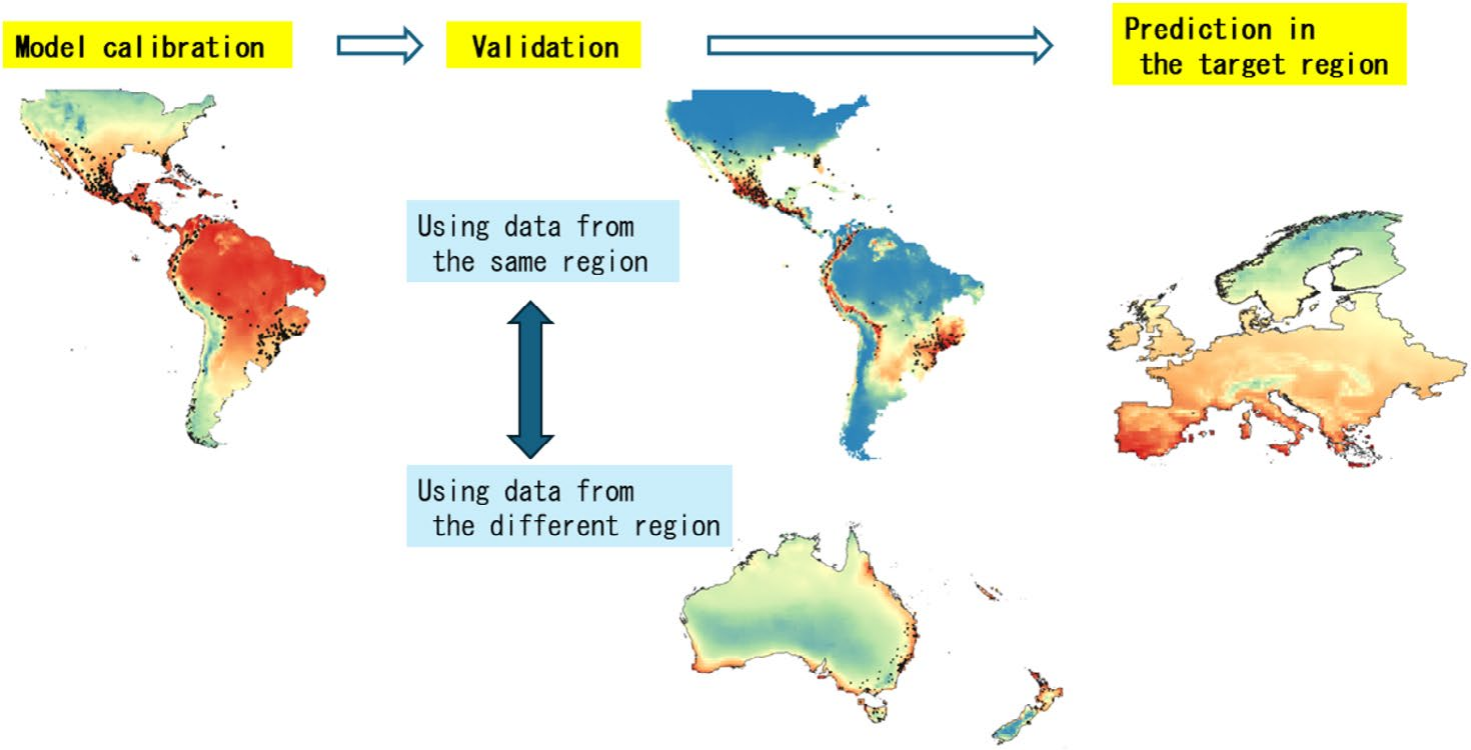
Different types of model validation.

Ideally validation should be conducted using test data from distinct geographical regions or time periods. However, most studies have evaluated model performance by randomly partitioning occurrence data into calibration and test datasets (Radosavljevic and Anderson 2014). Given that species distribution data typically exhibit spatial autocorrelation (Bahn and McGill 2013), this practice can lead to inflated evaluator values (Hijmans 2012; Bahn and McGill 2013; Radosavljevic and Anderson 2014). Therefore, robust model validation requires appropriate testing schemes: for example, internal random holdout or spatially independent test datasets (Figure 1, Table 1).

**TABLE 1:** Relationship between test dataset and training dataset.

Test dataset type	Distribution data	Environmental data
Internal random holdout test dataset	Sourced from the same region Randomly partitioned from the training data	Same as the training data
Spatially independent test dataset	Sourced from the different region	Sourced from the different region

In addition to testing schemes, suitable evaluators are essential for testing the predictive performance of models. Previous studies have employed quantitative performance measures to evaluate model quality (Radosavljevic and Anderson 2014). The area under the receiver operating characteristic curve (AUC) serves as the most widely used evaluator (Lobo et al. 2008; Lawson et al. 2014). This approach mitigates the supposed subjectivity in the threshold selection process, wherein continuous probability‐derived scores are converted into a binary presence‐absence variable, by summarizing the overall model performance across all possible thresholds (Lobo et al. 2008). However, Lobo et al. (2008) stated that the AUC had several important drawbacks when applied to species distribution modeling, and most importantly that the spatial extent of the prediction greatly influenced the AUC values.

In this study, I aimed to identify the appropriate method for assessing the transferability of SDMs in validation, specifically in terms of test dataset and evaluator selection. The model evaluation ability of validation methods was examined using several completely independent datasets that are rarely used in other studies. Previous studies have advocated for non‐random cross‐validation to assess model transferability (Wenger and Olden 2012; Radosavljevic and Anderson 2014) rather than conventional random holdout cross‐validation. In non‐random cross‐validation, data are divided into multiple geographic regions, such that any geographical region used for validation differs from those used for training the model. Although previous studies applied this approach using datasets from the same region, this study employed completely independent datasets sourced from different continents to verify its advantage over internal random holdout cross‐validation. Although geographically distinct test datasets are generally considered ideal, other aspects may influence their utility. Therefore, this study examined the ability of two testing schemes employing different test dataset types (one using internal random holdout datasets and the other employing datasets sourced from different continents) to evaluate model transferability. These two dataset types represent opposite ends of the spectrum in terms of independence, with non‐random cross‐validation falling somewhere between the two; therefore, the findings in this study are also considered relevant for conducting non‐random cross‐validation. Additionally, this study considered the appropriate evaluator when using test datasets from distinct geographical regions. The values of the most widely used evaluator (i.e., AUC) have been shown to be considerably influenced by the geographical extent over which predictions are made, potentially increasing uncertainty in model validation when predictive performance in different regions is evaluated. Therefore, this study compared the AUC with alternative evaluators to determine a more viable approach.

## 2 Methods

### 2.1 Species

Model transferability was examined using three invasive plant species: *Oxalis latifolia* Kunth, *Digitaria sanguinalis* (L.) Scop., and *Amaranthus retroflexus* L. Biological invasions serve as unique large‐scale biogeographical experiments for assessing model transferability across diverse environmental contexts. These three species have established populations across multiple continents over an extended period, making them suitable candidates for assessing model transferability.

### 2.2 Predictions of Distribution

Predictions were made using the methods described below to compare different schemes and evaluators for assessing model transferability.

maxent software (version 3.4.4; Phillips et al. 2006, 2017) was used in this study. This software is extensively utilized for modeling species distributions (Elith et al. 2011). The models were calibrated using a regularization multiplier value of two, indicating twice as much regularization as the default setting, to avoid overfitting. The default settings were used for all calibrations except for the regularization multiplier.

Distribution data were obtained from the database of the Global Biodiversity Information Facility (GBIF). The details are provided in Appendix A.

The following eight variables from the CliMond database (Kriticos et al. 2012, 2014) were used as environmental data: the annual mean temperature, temperature seasonality, mean temperature of the warmest quarter, mean temperature of the coldest quarter, precipitation seasonality, precipitation of the wettest quarter, annual mean moisture index, and moisture index seasonality. The details are provided in Appendix B.

In this study, the geographical region to which a model is transferred is referred to as the target region. When SDMs are utilized to predict the distributions in new geographical regions, the distribution of the species in the target region is usually unknown. However, in this study, geographical regions with data on the species distribution were selected as target regions to examine the evaluation ability of testing schemes. For each species, three or four invaded regions were selected for distribution predictions: Europe, Africa, and Oceania for *O. latifolia* ; North America, South America, Europe, and Africa for *D. sanguinalis* ; and Europe, Africa, and East Asia for *A. retroflexus* . The global presence data of the three species (GBIF.org 2023a, 2023b, 2023c) indicate that the presence data for these species were recorded in or before 1970 in the primary areas of their post‐1970 distributions within each region. Based on this, it was assumed that their distributions had reached equilibrium, allowing for a reliable evaluation of model performances using data from these regions.

In this study, the geographical region from which the training data are sourced is referred to as the calibration region. The models were calibrated using data from various regional combinations to predict the distribution within the target region (Table 2). Model performance frequently varies based on the calibration region, allowing for the development of distinct performance models for the same target region. Data were exclusively sourced from native regions: America for *O. latifolia* , Europe for *D. sanguinalis* , and North America for *A. retroflexus* . Additionally, data from both native and invaded regions were aggregated into a single dataset. This mimics the actual model construction for predicting invasion potential in novel regions using data solely from the native region or a combination of the native and invaded regions. Data from the invaded regions utilized all combinations of three or four regions except for the target region. Therefore, three different calibration datasets were used for one target region in the case of *O. latifolia* and *A. retroflexus* , whereas eight different calibration datasets were employed for one target region in the case of *D. sanguinalis* . A total of 56 predictions were made.

**TABLE 2:** Distribution predictions for three species.

Species	Target region	Calibration regions
*Oxalis latifolia*	Oceania	America, America + Africa, America + Europe, America + Africa +Europe (4 combinations)
	Africa	America, America + Oceania, America + Europe, America + Oceania + Europe (4 combinations)
	Europe	America, America + Africa, America + Oceania, America + Africa + Oceania (4 combinations)
*Digitaria sanguinalis*	Oceania	Eu, Eu + Af, Eu + NA, Eu + SA, Eu + Af + NA, Eu + Af + SA, Eu + NA + SA, Eu + Af + NA + SA (8 combinations)
	Africa	Eu, Eu + Oc, Eu + NA, Eu + SA, Eu + Oc + NA, Eu + Oc + SA, Eu + NA + SA, Eu + Oc + NA + SA (8 combinations)
	North America	Eu, Eu + Af, Eu + Oc, Eu + SA, Eu + Af + Oc, Eu + Af + SA, Eu + Oc + SA, Eu + Af + Oc + SA (8 combinations)
	South America	Eu, Eu + Af, Eu + Oc, Eu + NA, Eu + Af + Oc, Eu + Af + NA, Eu + Oc + NA, Eu + Af + Oc + NA (8 combinations)
*Amaranthus retroflexus*	Oceania	North America, North America + East Asia, North America + Europe, North America + East Asia + Europe (4 combinations)
	East Asia	North America, North America + Oceania, North America + Europe, North America + Oceania + Europe (4 combinations)
	Europe	North America, North America + Oceania, North America + East Asia, North America + Oceania + East Asia (4 combinations)

*Note:* In the “calibration region” column of the row corresponding to “ *Digitaria sanguinalis* ”, the region names are abbreviated: Af, Africa; Eu, Europe; NA, North America; Oc, Oceania; SA, South America.

### 2.3 Evaluation of Prediction Results

Data from the target regions were used to evaluate the results of predictions described in Section 2.2. When SDMs are utilized to predict the distributions in new geographical regions, the distribution of the species in the target region is usually unknown. However, in this study, geographical regions with data on the distribution of the species were selected as target regions to examine the evaluation ability of testing schemes. Three threshold‐independent evaluators were used: AUC, continuous Boyce index (CBI), and correlation coefficient with the predictions from the model calibrated in the predicted region (RWIP).

Modeling methods, such as maxent, produce continuous‐valued outputs, necessitating a threshold to predict presence or absence. Evaluators, including sensitivity, specificity, and true skill statistics (TSS), are contingent upon the discrimination thresholds. The AUC circumvents the assumed subjectivity in the threshold selection process by summarizing the overall model performance over all possible thresholds. The AUC is widely used for this reason (Lobo et al. 2008). Lawson et al. (2014) systematically reviewed 100 SDM studies that employed single‐number performance evaluators. Most studies have used threshold‐independent evaluators, primarily the AUC. Although 50% of the studies employed threshold‐dependent evaluators, most of these investigations utilized threshold‐independent evaluators.

Although the AUC relies on presence/absence information, occurrence data derived from specimens or observations do not provide absence information (such occurrence data are referred to as presence‐only data). Presence data were obtained from the GBIF database, and the cells of climate data that contained presence data were defined as presence and the cells that did not as absence to calculate the AUC for predictions in the target regions.

A literature review indicated that over half of research employing SDMs utilized presence‐only occurrence data for model construction (Guillera‐Arroita et al. 2015). Boyce et al. (2002) proposed an evaluator of predictions from models using presence‐only occurrence data (Boyce index), and Hirzel et al. (2006) further refined the algorithm. The Boyce index operates on the premise that if a model accurately predicts the distribution, the relative frequency of the presence points should increase with the prediction value of the model. Boyce et al. (2002) divided both the presence and background points into approximately 10 bins according to their prediction values. Hirzel et al. (2006) introduced a moving‐window approach (characterized by successive, partially overlapping windows). They defined a habitat suitability map composed of cells with prediction values ranging from 0 to 1. For each moving window, three ratios were calculated: the ratio of cells containing presence data to that in the entire map (P); the ratio of all cells within the moving window relative to the total number of cells in the entire map (E); and the ratio of these two values (P/E). Spearman's rank correlation coefficient between the P/E ratio and median of the prediction value for each moving window was subsequently calculated. This value was referred to as the “continuous Boyce index” (CBI). This evaluator was used, and a 0.1 wide window was moved in increments of 0.01 based on the descriptions and figures in the study. To calculate this evaluator, all the moving windows with at least one cell were used.

The third evaluator, RWIP, is, as far as I know, a novel evaluator. When using the RWIP to evaluate the prediction result for region X from model A, model B is first calibrated using data from region X. Subsequently, the distribution in region X is predicted using model B. This converts the binary distribution data into the Maxent output, which is continuous data. Maxent excels at predicting regional distributions based on data from the corresponding region (Elith et al. 2006). The evaluation of prediction result for region X from model A is carried out by comparing this prediction result with the prediction result for region X from model B. Maxent generates a prediction value for each cell within the environmental data grid. The Pearson correlation coefficients between the pair of predictions are calculated using cell‐by‐cell prediction values. (In this study, correlation coefficient refers to Pearson correlation coefficient.)

Methods that evaluate two predictions, similar to the RWIP, can be used to measure the niche overlap between two species. The evaluators of niche overlap, such as Schoener's D, Hellinger distance, and modified Hellinger distance (I) (Warren et al. 2008) are based on differences in prediction values for each cell or on differences in the square roots of prediction values for each cell of the two predictions. Using presence‐only distribution data for model calibration yields predictions of the relative relationships between distribution probabilities across cells rather than the absolute distribution probabilities. Consequently, a correlation coefficient was used instead of the difference between the prediction values of two predictions.

The predictive performance of SDMs is frequently assessed using evaluator classifications. In this study, the AUC was used to examine the evaluation ability of the conventional method, with values of ≥ 0.8 rated as “good”, 0.7–0.8 as “fair”, and < 0.7 as “poor”. For the CBI, values of ≥ 0.7 were rated as “good,” following Petitpierre et al. (2017), who considered this threshold as a key criterion for model transferability. For the RWIP, values of ≥ 0.7 were provisionally rated as “good,” aligning with conventional correlation coefficient classifications.

### 2.4 Examining the Evaluation Ability for Testing on Internal Random Holdout Datasets

Four‐fold random holdout cross‐validation was implemented using the training data of each model described above, and the average evaluator values and evaluator values of the predictions in the target regions were compared. This comparison assessed whether models rated as “good” in random holdout cross‐validation maintained the same rating when predicting in the target regions. Additionally, the correlation coefficients and *p*‐values between the evaluator values of random holdout cross‐validation and prediction in the target regions were calculated. (In this study, the *p*‐value was calculated using the cor.test function in the statistical software R). For this comparison, the AUC and CBI were used as evaluators. When selecting the “crossvalidate” setting, maxent randomly partitions presence data and calculates the AUC using background data rather than absence data. The CBI was calculated using partitioned presence and background data. Because the predicted region overlapped with the model calibration region in the cross‐validation, the RWIP was not calculated.

Additionally, whether novel climate affects the predictive performance was inferred from the correlation coefficient and *p*‐value between the ratios of the novel climate areas and the evaluator values in the target region. The details are provided in Appendix D.

### 2.5 Examining the Evaluation Ability for Testing on Spatially Independent Datasets

In this study, the geographical region from which the test data are sourced is referred to as the test region. (When internal random holdout test data are used for model validation, the test region is the same as the calibration region.)

When SDMs are utilized to predict the species distribution in a new geographical region, the distribution in the target region is usually unknown. Since model validation requires test data that include distribution data, the test region is different from the target region. Consequently, in such studies, the predictive performance of the models in the target regions is estimated based on evaluator values for the predictions in the test region.

Ideally, test data should be sourced from a region spatially independent from the calibration region (Radosavljevic and Anderson 2014; Yates et al. 2018). Jiménez‐Valverde et al. (2011) suggested model validation using data from different geographical regions for invasive species risk assessment. Although spatially independent test datasets are generally considered ideal, other aspects may influence their utility.

To examine the evaluation ability for testing on spatially independent datasets, paired predictions from the same models in different geographical regions were compared. For example, if a model is trained with data from America and is used to predict distribution in Oceania and Africa, the predictions for Oceania and for Africa are the paired predictions. A predictive model is validated by making a prediction using data from the test region and is then used to make a prediction in the target region. Consequently, the same model is used to make predictions for the two regions. Therefore, the relationship between abovementioned Oceania and Africa predictions is the same as the relationship between the validation prediction and the target region prediction. If the prediction result in validation is good, a good prediction will also be estimated in the target region, and vice versa. In this study, by examining the relationship between the paired predictions, the evaluation ability for testing on spatially independent datasets was evaluated.

Using the distribution predictions for the three species described above, 36 pairs of predictions were generated for two different regions employing the same model (Table A5 in Appendix E), and the evaluation values between the two predictions were compared for each pair. The evaluation ability for testing on spatially independent datasets was assessed by calculating the correlation coefficient between the higher and lower evaluator values for each pair. This comparison was conducted using the three evaluators described in Section 2.3.

## 3 Results

### 3.1 Predictions of Distribution

The evaluator values of the distribution prediction of the three species varied mainly based on the training data (Tables A1, A2, A3 in Appendix C, Figures S1–S3). The highest values for each species‐target region combination were 0.73–0.95 (with 9 out of 10 being ≥ 0.80) when the AUC was used, 0.83–1.00 (with 9 out of 10 being > 0.90) when the CBI was used, and 0.66–0.97 (with 9 out of 10 being > 0.70) when the RWIP was used. Based on these evaluation values, most predictions were rated as good. In contrast, the lowest values for each species‐target region combination were 0.53–0.92 (with 3 out of 10 being < 0.70) when the AUC was used, −0.43 to 0.93 (with 7 out of 10 being < 0.70) when the CBI was used, and −0.02 to 0.89 (with 7 out of 10 being < 0.70) when the RWIP was used. These evaluation values varied widely, with some predictions rated as poor.

**FIGURE 2:**
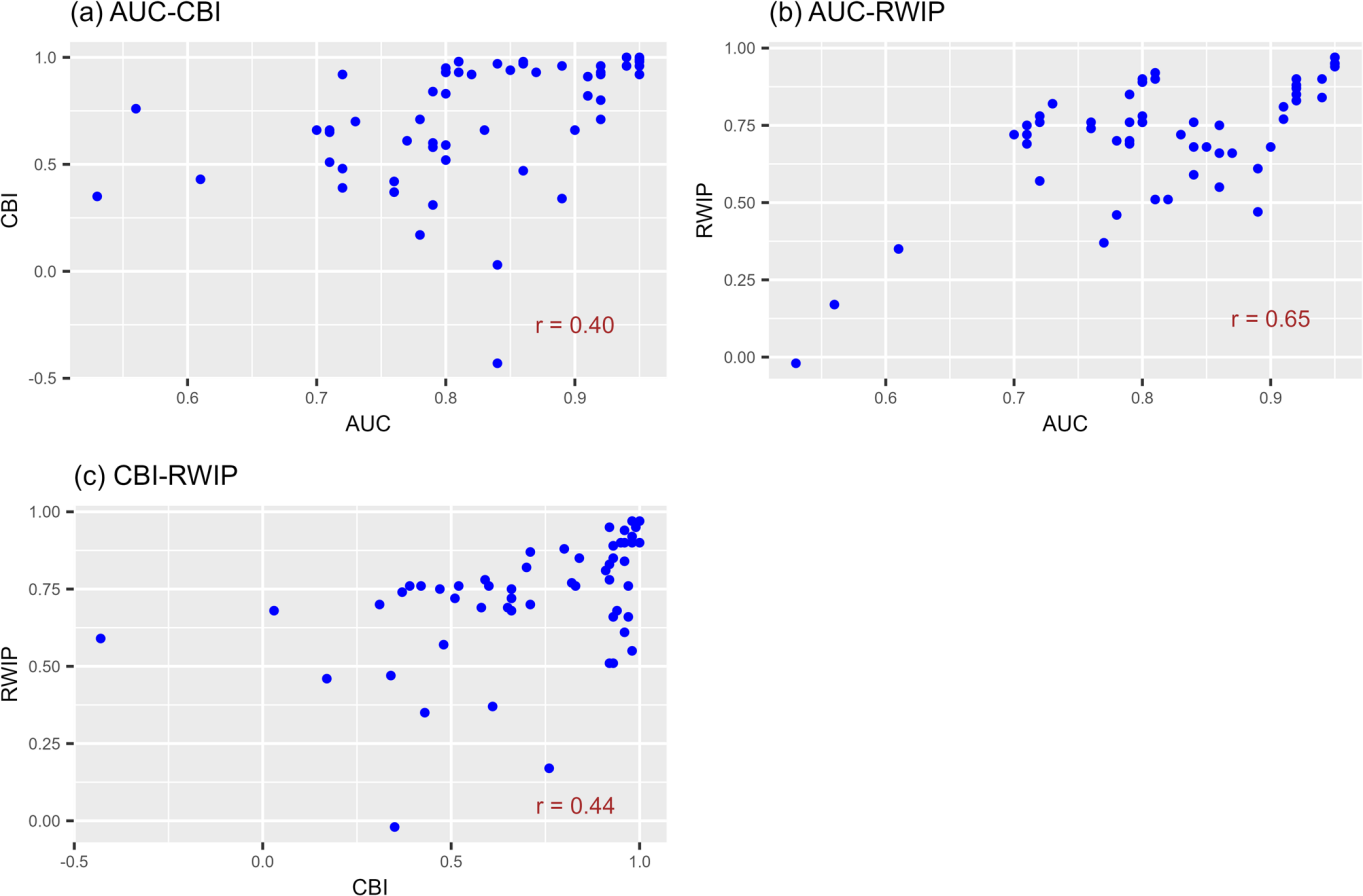
Relationship between the values of three evaluators (AUC, CBI, and RWIP) for the same distribution predictions. (a) Relationship between the values of AUC and CBI for the same distribution predictions. (b) Relationship between the values of AUC and RWIP for the same distribution predictions. (c) Relationship between the values of CBI and RWIP for the same distribution predictions. Each point in the scatter plots corresponds to one of the 56 predictions for three species ( *Oxalis latifolia* , *Digitaria sanguinalis* , and *Amaranthus retroflexus* ). The distribution predictions were made for the species in each invaded region using training data from different regional combinations. AUC, the area under the receiver operating characteristic curve; CBI, continuous Boyce index; and RWIP, correlation coefficient with the predictions from the model calibrated in the predicted region.

The correlation coefficients between the three evaluators for the same prediction were 0.40 between the AUC and CBI (*p* = 0.0024), 0.65 between the AUC and RWIP (*p* = 5.4 × 10^−8^), and 0.44 between the CBI and RWIP (*p* = 0.00073) (Figure 2). Although positive correlations were observed, the evaluations differed according to the evaluator.

### 3.2 Examining the Evaluation Ability for Testing on Internal Random Holdout Datasets

**FIGURE 3:**
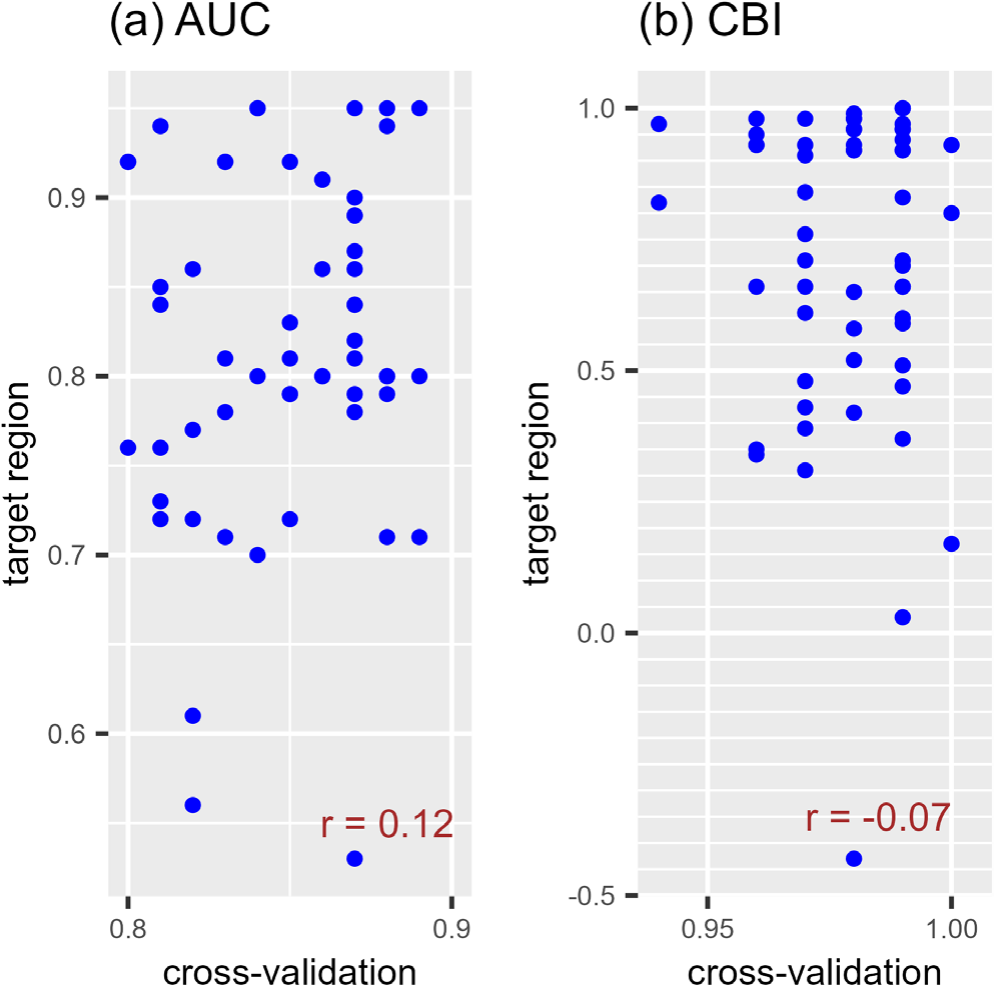
Relationship of evaluator values between internal random holdout cross‐validations and predictions in the target region. (a) Relationship of AUC values between random holdout cross‐validations and predictions in the target region. (b) Relationship of CBI values between random holdout cross‐validations and predictions in the target region. Each point in the scatter plots corresponds to one of the 56 predictions for three species ( *Oxalis latifolia* , *Digitaria sanguinalis* , and *Amaranthus retroflexus* ). The distribution predictions were made for the species in each invaded region using training data from different regional combinations. The *x*‐axis is the average evaluator value of internal random holdout cross‐validation, while the *y*‐axis is the evaluator value of the prediction in the target region. AUC, the area under the receiver operating characteristic curve; and CBI, continuous Boyce index.

The average evaluator value for the random holdout cross‐validation with the training dataset of each model was 0.80–0.89 and 0.94–1.00 when the AUC and CBI were used, respectively (Figure 3, Table A4 in Appendix C). All models were rated as good, whereas the evaluator values for prediction in the target regions varied widely (AUC, 0.53–0.95 and CBI, −0.43 to 1.00). Therefore, internal random holdout cross‐validation was unable to distinguish whether the predictive performance of the models was good or poor in the target regions. The correlation coefficient between the average evaluator values from the random holdout cross‐validation and the evaluator values of the predictions in target regions was 0.12 when the AUC was used (*p* = 0.36) and −0.07 when the CBI was used (*p* = 0.63). No correlation was observed.

**FIGURE 4:**
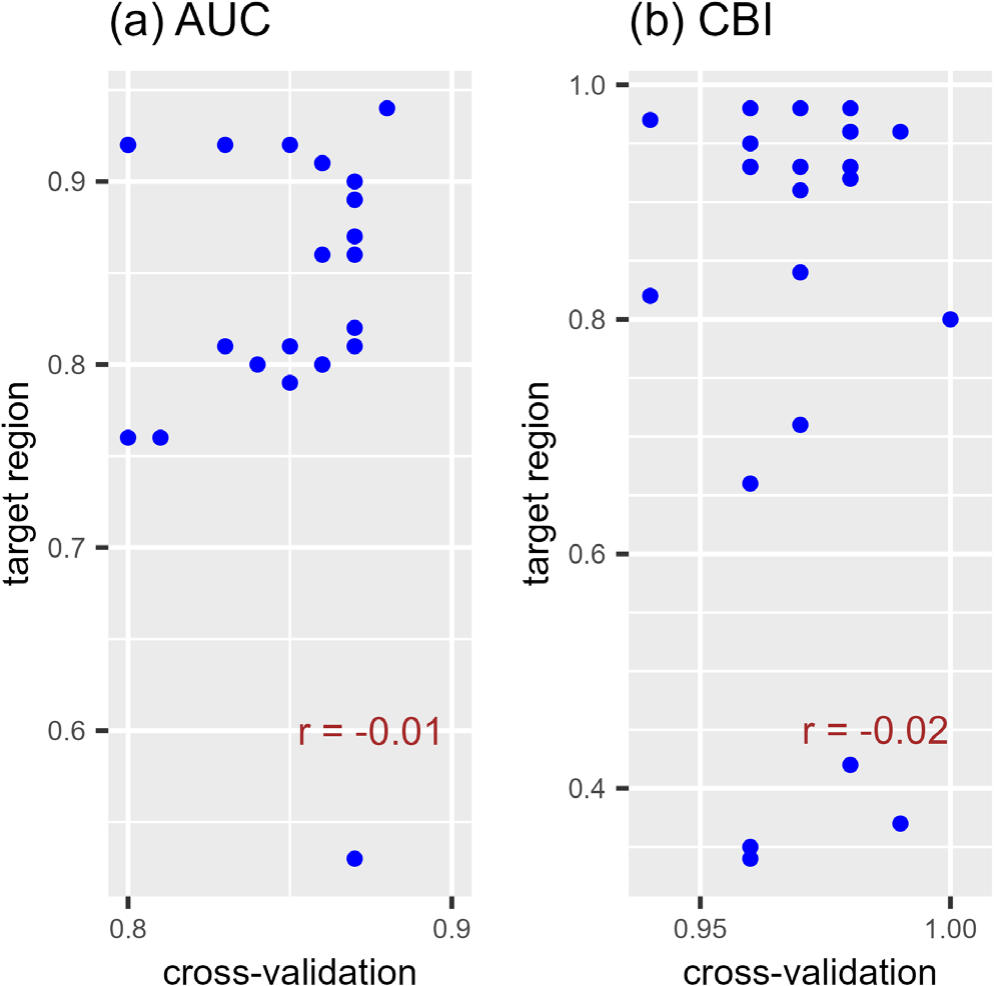
Relationship of evaluator values between internal random holdout cross‐validations and predictions in the target region (the prediction for two species). (a) Relationship of AUC values between random holdout cross‐validations and predictions in the target region. (b) Relationship of CBI values between random holdout cross‐validation and predictions in the target region. Each point in the scatter plots corresponds to one of the 24 predictions for two species ( *Oxalis latifolia* and *Amaranthus retroflexus* ). The distribution predictions were made for the species in each invaded region using training data from the different combinations of regions. The *x*‐axis is the average evaluator value of internal random holdout cross‐validation, while the *y*‐axis is the evaluator value of the prediction in the target region. AUC, the area under the receiver operating characteristic curve; and CBI, continuous Boyce index.

Additionally, whether novel climate affects the predictive performance was inferred from the correlation coefficient between the ratios of the novel climate areas and the evaluator values in the target region (see Appendix D). These correlations did not suggest that including novel climate areas had a negative effect on the prediction accuracy for the distribution of the two species aside from *D. sanguinalis* . The distribution predictions for the two species exhibited considerable variability in evaluator values within the target regions (AUC, 0.53–0.94 and CBI, 0.34–0.98). Therefore, internal random holdout cross‐validation was unable to distinguish whether the predictive performance of the models was good or poor in the target regions. Furthermore, the correlation coefficient between the average evaluator values from the random holdout cross‐validation and evaluator values of the predictions in target regions was −0.01 when the AUC was used (*p* = 0.96) and −0.02 when the CBI was used (*p* = 0.91). No correlation was observed (Figure 4).

### 3.3 Examining the Evaluation Ability for Testing on Spatially Independent Datasets

**FIGURE 5:**
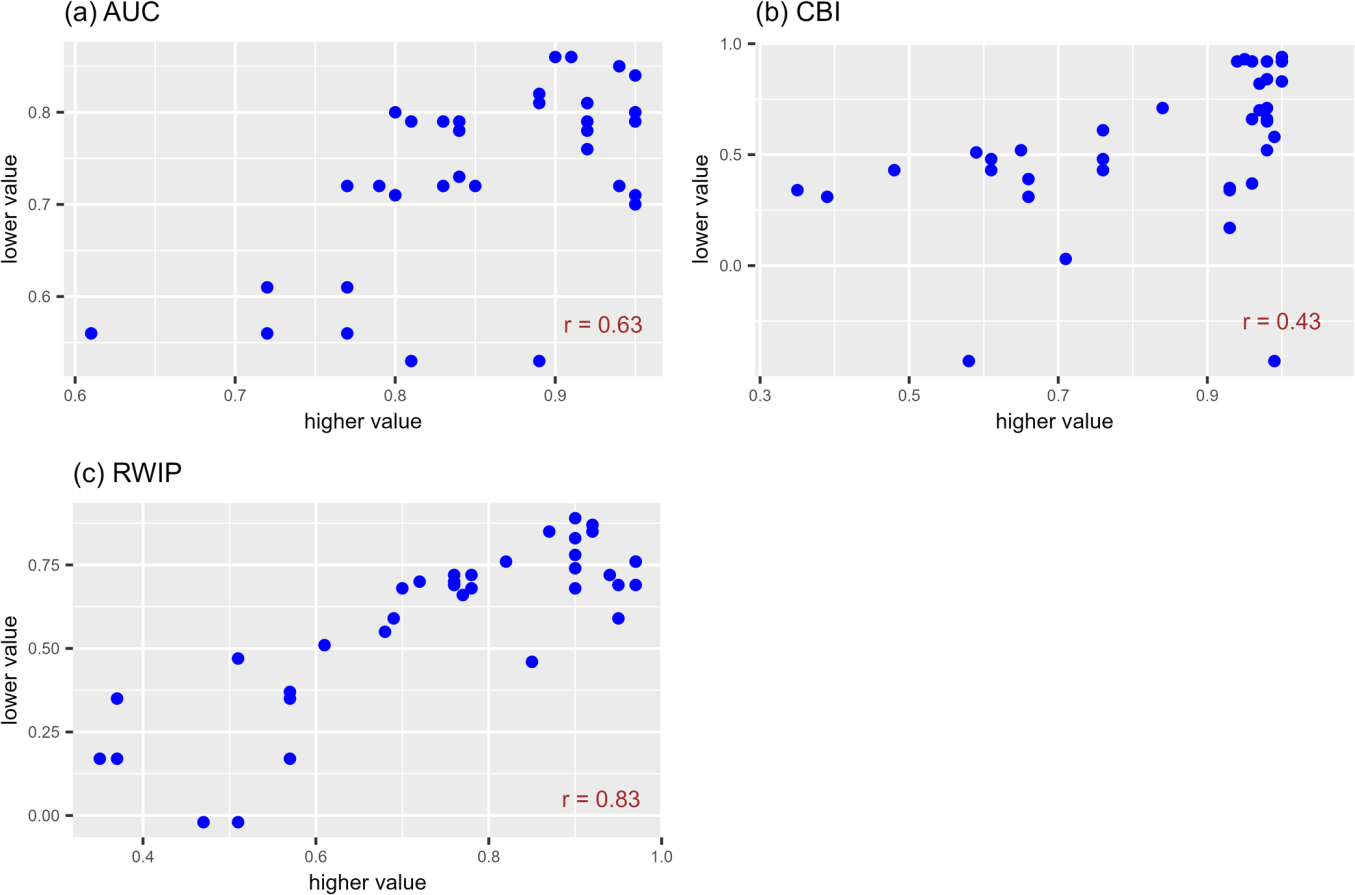
Relationship of evaluator values between paired distribution predictions from the same models in different regions. (a) Relationship of AUC values between paired distribution predictions from the same models in different regions. (b) Relationship of CBI values between paired distribution predictions from the same models in different regions. (c) Relationship of RWIP values between paired distribution predictions from the same models in different regions. Each point in the scatter plots corresponds to one of the 36 pairs of predictions for three species ( *Oxalis latifolia* , *Digitaria sanguinalis* , and *Amaranthus retroflexus* ). The distribution predictions were made for the species in each invaded region using training data from the different combinations of regions. The *x*‐coordinate of each point is the higher evaluator value, while the corresponding *y*‐coordinate is the lower evaluator value of the paired prediction. AUC, the area under the receiver operating characteristic curve; CBI, continuous Boyce index; and RWIP, the correlation coefficient with the predictions from the model calibrated in the predicted region.

The correlation coefficients for the paired predictions across various regions using the same models were 0.63 (*p* = 3.9 × 10^−5^), 0.43 (*p* = 0.0085), and 0.83 (*p* = 2.5 × 10^−10^) when the AUC, CBI, and RWIP were used, respectively (Figure 5, Table A5 in Appendix E). A strong correlation was observed exclusively when the RWIP was used. The relationship between the paired predictions mirrored that between the predictions using the test datasets and predictions in the target region. Therefore, employing the RWIP maintained a relatively low uncertainty when testing the predictive performance of the models on spatially independent datasets.

## 4 Discussion

The distribution predictions for the three species exhibited predominantly good accuracy in identifying the optimal predictions for each species‐target region combination. In such cases, selecting models with high predictive performance in target regions substantially enhances prediction reliability. However, when SDMs are applied to predicting species distribution, the species distribution in the target region is often unknown. Therefore, selecting appropriate test datasets and evaluators is essential for assessing model transferability.

### 4.1 Testing Schemes (Internal Random Holdout Test Datasets or Spatially Independent Test Datasets)

**FIGURE 6:**
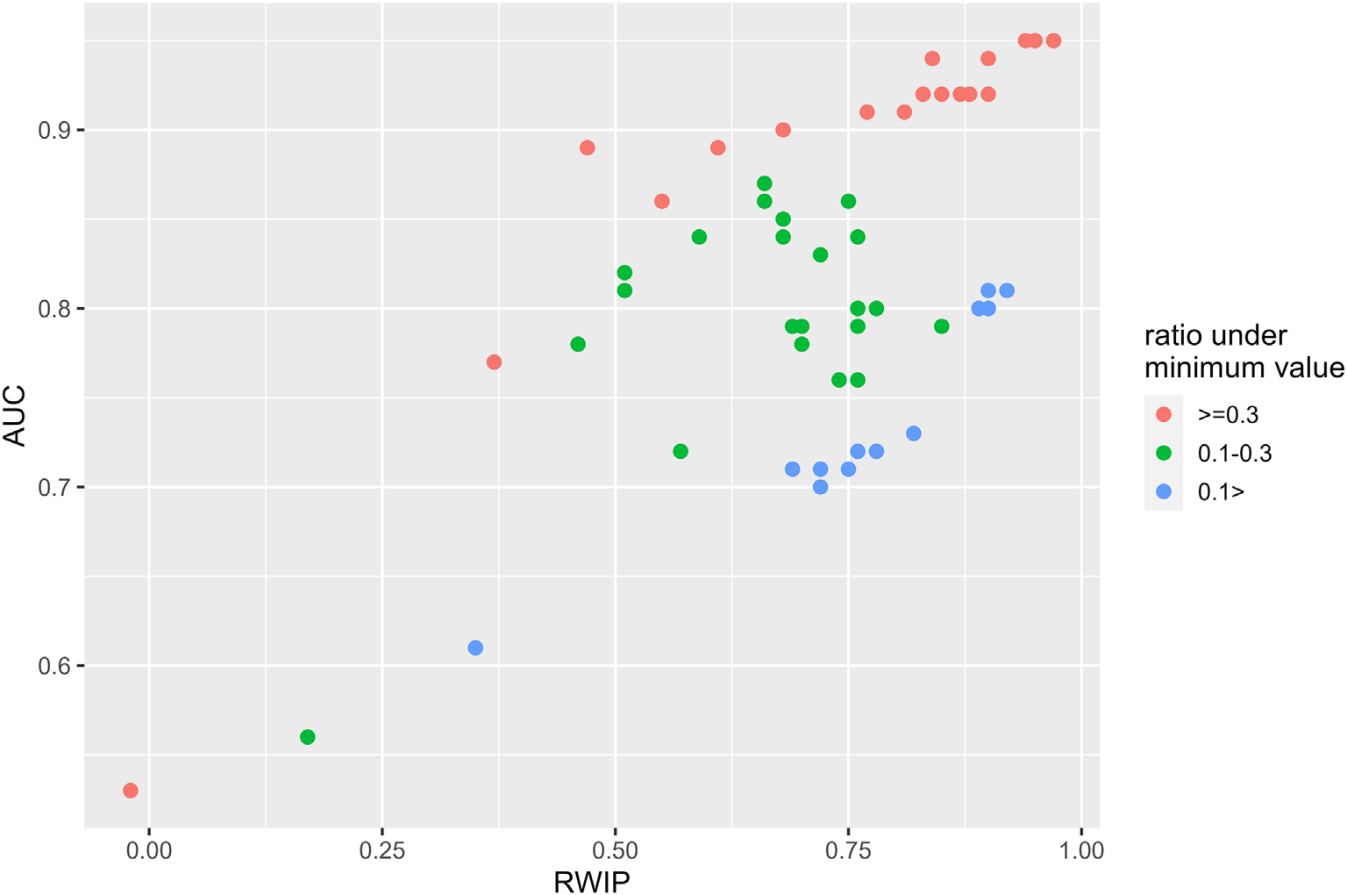
Relationship between two evaluator values for the same distribution prediction and the ratio of unsuitable area. Each point in the scatter plot corresponds to one of the 56 predictions for three species ( *Oxalis latifolia* , *Digitaria sanguinalis* , and *Amaranthus retroflexus* ). The distribution predictions were made for the species in each invaded region using training data from the different combinations of regions. The x‐axis is the value of correlation coefficient with the predictions from the model calibrated in the predicted region (RWIP), while the y‐axis is the value of the area under the receiver operating characteristic curve (AUC). The color of each symbol in the scatter plot represents a class determined by the ratio of the unsuitable area. Each species‐target region combination was classified as follows: For each prediction, the lowest prediction value among the cells with presence data was set as a threshold, and the ratio of the number of the cells whose prediction values were under this threshold to all cells in the region was calculated. Thereafter, the highest value in each combination was taken as the representative value of the combination. For the predictions with the red, green, and blue symbols, the values are ≥ 0.3, 0.1–0.3, and < 0.1, respectively.

In this study, two testing schemes differing in terms of the type of test dataset were compared. In internal cross‐validations with random holdout test datasets, all models were rated as good; however, the evaluator values for predictions in the target regions ranged widely and included instances of poor predictions. When the AUC was used, fewer predictions were rated as poor than when the CBI was used. However, owing to the drawback of the AUC, as described later, the AUC values of some predictions were inflated (see Figure 6). This indicates that the evaluator values derived from validations using the internal random holdout test datasets are not suitable for estimating the predictive performance of models in the target regions because this approach fails to distinguish whether the model has good or poor predictive performance in these regions.

Some studies have shown that testing models using data from the same region as the training data can inflate the evaluator values by applying data treatments addressing spatial autocorrelation (Hijmans 2012) or comparing predictions with different data partitioning (Bahn and McGill 2013; Radosavljevic and Anderson 2014). The findings of these studies suggest that the evaluation values are inflated due to the use of random holdout test datasets, resulting in a considerable reduction in the evaluation ability of the model for testing.

In contrast to these studies, which each employed data from the same region, Petitpierre et al. (2017) used completely independent data sourced from different continents. They compared the strategies for selecting predictors to maximize the transferability of SDMs on a cross‐continental scale. The distributions of 50 Holarctic plant invaders were predicted in both native and invaded Holarctic regions and Australia using 10 distinct strategies for predictor selection. The results showed that the internal cross‐validation with random holdout test datasets was inadequate to fully assess the transferability of the models to a different region. In the predictions for native regions, the average evaluator values from random holdout cross‐validations indicated that most CBI values for the combinations of 50 species and 10 predictor selection strategies were > 0.9, with a minimum value of 0.75. However, predictions for invaded regions varied widely, with the minimum value recorded at −0.96 (Petitpierre et al. 2017). Similarly, in the present study, CBI values for predictions in target regions showed a much broader range, including substantially lower values than those obtained from random holdout cross‐validation.

A positive correlation was observed between the evaluator values of the paired distribution predictions from the same models across different regions. The relationship between paired predictions mirrored that between predictions derived from test datasets and predictions in the target region. This indicates that the evaluator values derived from predictions using the spatially independent test datasets can be used to estimate the predictive performance of models in target regions. However, the correlation strength varied depending on the evaluator used, as discussed in the next section.

### 4.2 Evaluators in Testing on Spatially Independent Datasets

To examine the evaluation ability for testing on spatially independent datasets, paired distribution predictions from the same models in different regions were compared. The correlation coefficients between the evaluator values of the paired distributions were 0.63, 0.43, and 0.83 when the AUC, CBI, and RWIP were used, respectively. This indicates that using the AUC or CBI increases the uncertainty of the model evaluation, whereas using the RWIP keeps the uncertainty relatively low.

Lobo et al. (2008) identified a critical drawback of the AUC: the area of the region over which predictions were made significantly affected the AUC values. Incorporating more absence points that are geographically distant from presence points increases the accuracy of predicted absences and improves the AUC value. When calculating the AUC values in this study, the cells of climate data that contained presence data were defined as presence, while the cells that did not contain presence data were defined as absence. Therefore, the AUC values varied based on the ratio of absence cells that were environmentally distant from presence cells. For example, the lowest maxent output value among presence cells serves as a threshold indicative of a suitable environment for a species. The ratio of cells with maxent output values below this threshold relative to the total number of cells in the region varied depending on the species‐region combinations and prediction model used (Tables A1, A2, A3 in Appendix C). For each species‐region combination, the maximum value of this ratio was considered the representative value. Figure 6 presents a scatterplot illustrating the relationship between the RWIP and AUC, with the color of each symbol corresponding to the ratio classification. This scatter plot illustrates that similar RWIP values correspond to high AUC values when the ratio is high and vice versa. Therefore, comparing the predictions across various regions based on the AUC introduces uncertainty. This could be one reason why the correlation coefficient was lower when the AUC was used than that when the RWIP was used to compare paired predictions from the same models in different regions.

**FIGURE 7:**
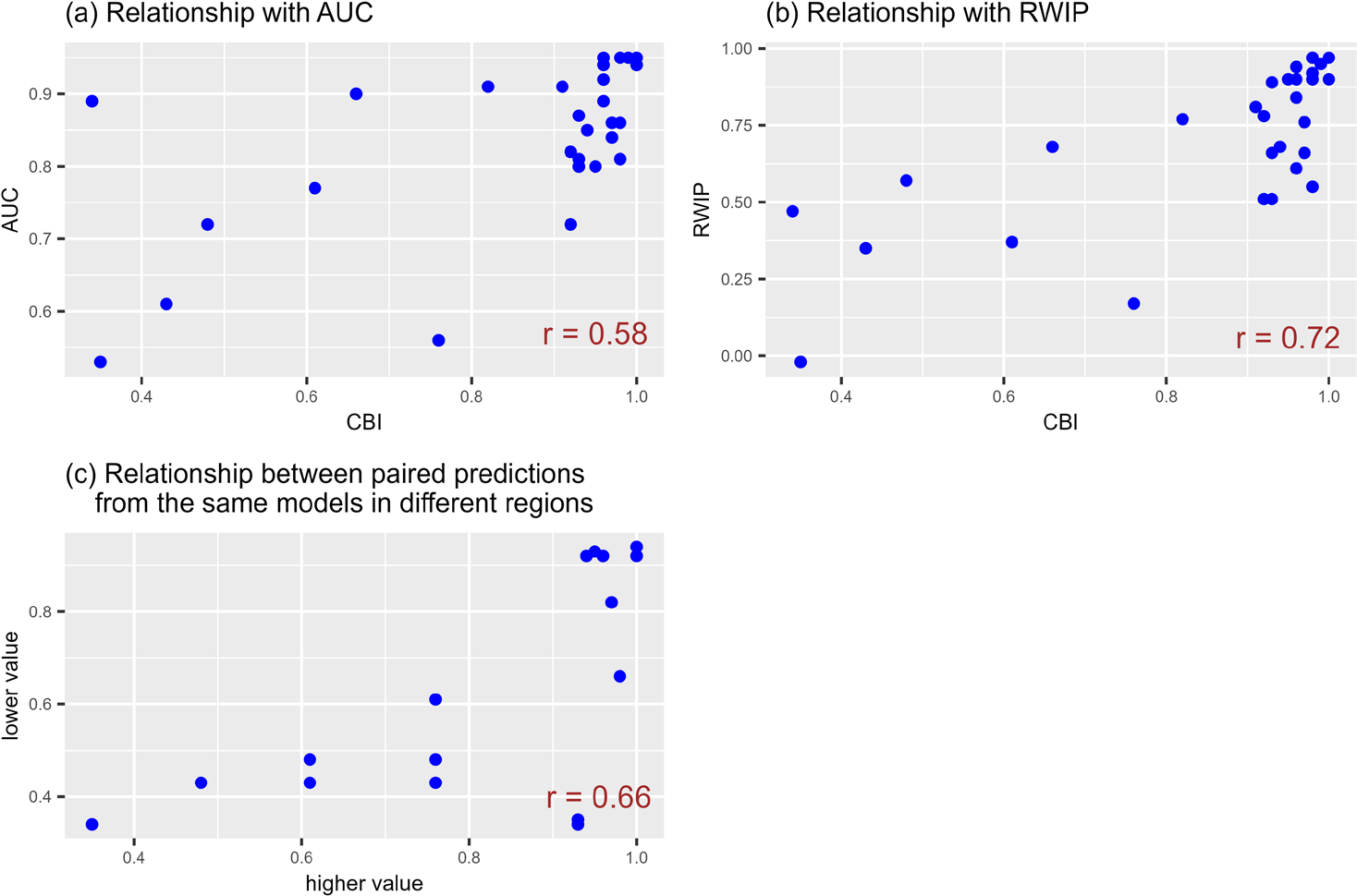
Continuous Boyce index (CBI) for predictions whose highest value moving window contained presence data; the relationships with other evaluators and between paired distribution predictions from the same models in different regions. A total of 56 distribution predictions were made for three species ( *Oxalis latifolia* , *Digitaria sanguinalis*, and *Amaranthus retroflexus* ) in each invaded region using training data from the different combinations of regions. In 30 of the 56 predictions, the moving window with the highest prediction value (among moving windows with at least one cell) contained presence data. (a) Relationship with AUC. Each point corresponds to one of the 30 predictions; the *x*‐axis is the CBI value; the *y*‐axis is the value of the area under the receiver operating characteristic curve (AUC). (b) Relationship with RWIP. Each point corresponds to one of the 30 predictions; the *x*‐axis is the CBI value; the *y*‐axis is the value of the correlation coefficient with the predictions from the model calibrated in the predicted region (RWIP). (c) Relationship of CBI values between paired predictions from the same models in different regions. Each point corresponds to one of the paired predictions from the same models in different regions made from the 30 predictions; the *x*‐coordinate of each point is the higher evaluator value, and the corresponding *y*‐coordinate is the lower evaluator value of the paired predictions.

The CBI was calculated from the values of each moving window. The distribution of the number of cells in each moving window varies depending on the predictions. Some predictions indicate that the number of cells within the moving windows exhibiting high prediction values is minimal. Because presence‐only distribution data were used, the unavailability of presence data in a cell could have been due to inadequate specimen sampling or observation. A very low number of cells increases the likelihood that presence data are unavailable across the involved cells. The absence of presence data within a moving window exhibiting a high prediction value results in a considerable decrease in the CBI value. Based on the results of this study, the likelihood of such cases is elevated in predicting distribution in a region other than the calibration region. In a single instance among the 116 predictions in the random holdout cross‐validation (29 datasets × 4), the moving window exhibiting the highest prediction value did not contain any cells with presence data (the number of cells in the moving window was one). However, in 26 of the 56 predictions in the target regions, the moving window exhibiting the highest prediction value (among those containing at least one cell) lacked any cells with presence data (the number of all cells in the moving window and the percentage relative to the entire region were 1–10 and 0.01%–0.19%, respectively) (Tables A1, A2, A3 in Appendix C). In 21 of these predictions, two or more moving windows from the one with the highest value lacked any cells with presence data (the number of all cells in each of these moving windows and the percentage to the entire region were 1–28 and 0.01%–0.45%, respectively). Excluding predictions where the highest‐value moving window lacked any cells with presence data from the target region predictions led to an increase in the correlation coefficient of the CBI with the AUC and RWIP from 0.40 to 0.58 (*p*‐value: from 0.0024 to 0.00082) and 0.44 to 0.72 (*p*‐value: from 0.00073 to 5.9 × 10^−6^), respectively. Additionally, the correlation coefficient between the paired predictions from the same models in different regions increased from 0.43 to 0.66 (*p*‐value: from 0.0085 to 0.0052) (Figure 7).

The AUC and RWIP do not consider the actual prediction values; instead, they focus on the relative relationships between the prediction values. Even if all prediction values were very high or very low, they did not affect the AUC and RWIP values. However, the CBI did not exhibit this drawback. As previously described, a moving window with a high prediction lacking presence data results in a considerable decrease in the CBI value. Conversely, even when all the prediction values were high, the CBI value did not decrease substantially. Boyce et al. (2002) divided the test data into bins of equal sample sizes to calculate the original Boyce index. Because of the sensitivity of this index to the number of bins and their boundaries, Hirzel et al. (2006) introduced a method utilizing moving windows to overcome this limitation. This modification enables the evaluator to assess the distribution of observed presences across the continuous gradient of prediction values. However, depending on the prediction and presence data used for model evaluation, the distribution of the number of cells in each moving window must be considered.

Although the RWIP outperformed the AUC and CBI in this study (see Section 3.3), the RWIP functions as a threshold‐independent evaluator; therefore, thresholds must be selected to assess the potential for species establishment or spread in the subsequent step. Additionally, some uncertainty still remains in evaluating model transferability. Ideally, a model rated as “good” during validation should also be rated as “good” when predicting the distribution in the target region. In 24 of the 36 paired distribution predictions generated by applying the same model to different regions, the higher RWIP values of each pair were ≥ 0.7. However, in 9 of these 24 paired distribution predictions, the corresponding lower RWIP values were < 0.7, with a minimum value of 0.46. If the higher RWIP values of each pair reflect model validation and the lower RWIP values represent actual predictive performance in target regions, these nine models will fail validation when using a ≥ 0.7 threshold. To further improve the evaluation ability of model transferability, future research should investigate the effects of various test regions on evaluation reliability using a broader dataset.

This study employed datasets from different continents to ensure spatially independent test datasets. However, target species distributions are often limited. In such cases, non‐random cross‐validation is recommended to assess model transferability (Wenger and Olden 2012; Radosavljevic and Anderson 2014). The findings of this study suggest that evaluator selection may contribute to the uncertainty of model evaluation in non‐random cross‐validation. Therefore, further comparisons of the evaluation ability of evaluators are required to refine non‐random cross‐validation methodologies.

## 5 Conclusions

This study verified that internal cross‐validation using random holdout test datasets was insufficient for reliably distinguishing between models with good or poor predictive performance in target regions. This finding is consistent with that of previous research. Furthermore, when testing on spatially independent datasets, the AUC and CBI introduced greater uncertainty in model evaluation, whereas the RWIP maintained comparatively lower uncertainty. These two findings demonstrate that the conventional approach (using random holdout test datasets and the AUC) cannot reliably assess model transferability. Conversely, using spatially independent test datasets and the RWIP provides a more robust and reliable alternative.

This study implemented a cross‐continental model evaluation strategy, representing the highest achievable level of spatial independence relative to conventional random holdout validation. Given the logistical and biological constraints associated with compiling such datasets, its broader application can be limited. However, the findings of this study offer valuable insights for improving model transferability assessments through other methods, such as non‐random cross‐validation. In particular, the RWIP could serve as a robust evaluator in non‐random cross‐validation frameworks.

## Author Contributions

**Takayuki Matsui:** conceptualization (lead), data curation (lead), formal analysis (lead), investigation (lead), methodology (lead), resources (lead), software (lead), validation (lead), visualization (lead), writing – original draft (lead).

## Funding

The author has nothing to report.

## Conflicts of Interest

The author declares no conflicts of interest.

## Supporting information


**Figure S1:** Distribution predictions for *Oxalis latifolia* .
**Figure S2:** Distribution predictions for *Digitaria sanguinalis* .
**Figure S3:** Distribution predictions for *Amaranthus retroflexus* .

## Figures and Tables

**FIGURE A1:**
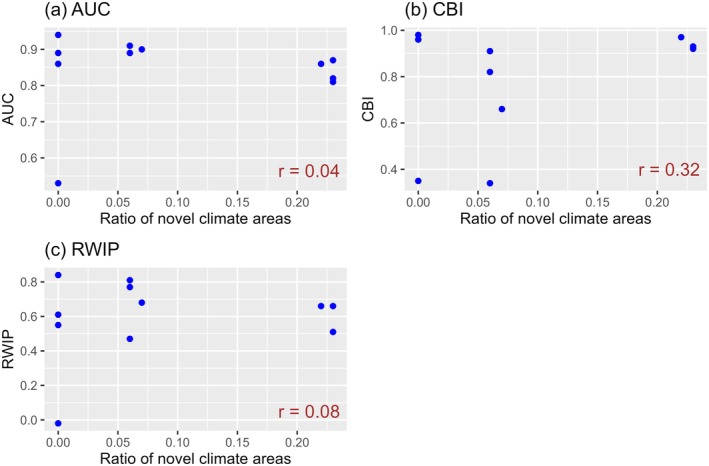
Relationship between ratio of novel climate areas and evaluator values (species: *Oxalis latifolia* ). Ratio of novel climate areas: Ratio of the number of cells exhibiting negative values in the multivariate environmental similarity surface (MESS) generated by Maxent. (a) Relationship between ratio of novel climate areas and AUC values. AUC, The area under the receiver operating characteristic curve. (b) Relationship between ratio of novel climate areas and CBI values. CBI, Continuous Boyce index. (c) Relationship between ratio of novel climate areas and RWIP values. RWIP, Correlation coefficient with the predictions from the model calibrated in the predicted region.

**FIGURE A2:**
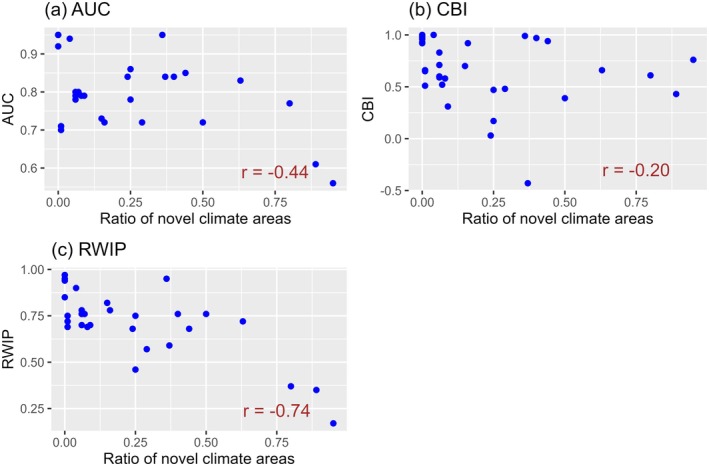
Relationship between ratio of novel climate areas and evaluator values (species: *Digitaria sanguinalis* ). Ratio of novel climate areas: Ratio of the number of cells exhibiting negative values in the multivariate environmental similarity surface (MESS) generated by Maxent. (a) Relationship between ratio of novel climate areas and AUC values. AUC, the area under the receiver operating characteristic curve. (b) Relationship between ratio of novel climate areas and CBI values. CBI, Continuous Boyce index. (c) Relationship between ratio of novel climate areas and RWIP values. RWIP, correlation coefficient with the predictions from the model calibrated in the predicted region.

**FIGURE A3:**
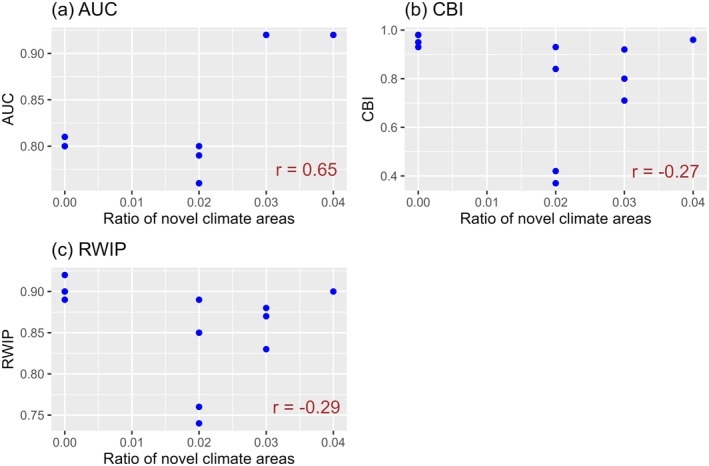
Relationship between ratio of novel climate areas and evaluator values (species: *Amaranthus retroflexus* ). Ratio of novel climate areas: Ratio of the number of cells exhibiting negative values in the multivariate environmental similarity surface (MESS) generated by Maxent. (a) Relationship between ratio of novel climate areas and AUC values. AUC, the area under the receiver operating characteristic curve. (b) Relationship between ratio of novel climate areas and CBI values. CBI, Continuous Boyce index. (c) Relationship between the ratio of novel climate areas and RWIP values. RWIP, correlation coefficient with the predictions from the model calibrated in the predicted region.

## Data Availability

The data that support the findings of this study are available from the corresponding author upon reasonable request.

## Appendix A

### Distribution Data

The occurrence data (global presence data) of the three species were downloaded from the Global Biodiversity Information Facility (GBIF) database between August and December 2023 (*O. latifolia* data were downloaded on August 26, *D. sanguinalis* data on October 21, and *A. retroflexus* data on December 15; GBIF.org 2023a, 2023b, 2023c, 2023d, 2023e, 2023f). The GBIF serves as the largest point of access for occurrence data derived from specimens and observations worldwide (Anderson et al. 2016).

Presence data with coordinate information from the regions of interest were extracted using QGIS (version 3) and shapefiles sourced from the global administrative areas (GADM) database. The GBIF database contains presence data, both with and without coordinate information. By comparing the two, if a country (or, in the case of the United States, Canada, Brazil, Russia, China, and Australia, an administrative division such as a state or province) possessed the latter but not the former, the country was excluded from both the calibration and target regions. This was because the models were constructed solely using presence data that included coordinate information; if the presence data used to build the model were not available in the area where the species were actually distributed, a typical sampling bias would occur.

To calibrate the models, the thinning described below was applied to the presence data, considering the difference in actual areas between the high‐ and low‐latitude regions within the geographic coordinate system. Phillips et al. (2006) presented the maximum entropy method (Maxent) for modeling the geographic distribution of species and outlined an idealized data model where the geographical study area was represented as a finite grid of equal‐sized cells (Phillips et al. 2017). However, when environmental data are used in grid cells based on a geographic coordinate system, the size of the cells varies with latitude. If the distribution density remains the same, the number of presence points is directly proportional to the area. To eliminate the effect of area differences due to latitude on the amount of presence data, the data were thinned according to the latitude of the area. First, the presence data of each region were divided into subsets at 1° latitude intervals according to the coordinate information of each presence point. Subsequently, the ratio of the longitudinal length corresponding to the latitude of each subset to that of the highest latitude subset for each species was calculated. The quantity of presence data in each subset was reduced in accordance with the abovementioned ratio by eliminating the presence data at equal intervals based on the longitude sequence number. These subsets were then combined and used as the dataset for each region to construct the models.

## Appendix B

### Environmental Data

Petitpierre et al. (2017) used bioclimatic variables from the CliMond database (Kriticos et al. 2012) to predict the distribution of 50 plant species across multiple continents and compared strategies for predictor selection. In the study, the following set of eight variables performed well on average: the annual mean temperature, temperature seasonality, mean temperature of the warmest quarter, mean temperature of the coldest quarter, precipitation seasonality, precipitation of the wettest quarter, annual mean moisture index, and moisture index seasonality. They advocate for including this set of variables to achieve robust projections of SDMs. Consequently, eight variables from CliMond (version 1.2; Kriticos et al. 2014) were used.

The CliMond database contains datasets at two resolutions: 10′ and 30′. A dataset with a 30′ resolution was selected. This selection pertains to the sampling bias correction method. Spatial sampling bias constitutes an important challenge associated with occurrence data derived from specimens or observations, such as the GBIF database, when used to construct SDMs (Anderson et al. 2016). Various factors can contribute to biases in SDMs, including survey location and spatial scale, data or specimen collection methods, and the storage and mobilization of collected data (Beck et al. 2014). Such biases can substantially distort SDM predictions (Fourcade et al. 2014; Beck et al. 2014).

Fourcade et al. (2014) evaluated five correction methods for the spatial sampling bias and found that simple systematic sampling performed well across the range of conditions tested. This method involved creating a grid of specified cell sizes and randomly sampling one presence record from each grid cell. When raster data are used as environmental data, maxent discards redundant records present within a single cell, which is the default setting. This is because, in this scenario, maxent interprets occurrence records as grid cells instead of points (Phillips et al. 2006). This is similar to applying simple systematic sampling. Beck et al. (2014) applied a similar method to presence data sourced from the GBIF database. The modeling region was divided into 100 × 100 km cells, from which a predefined maximum number (1–20) of records per cell was randomly selected (if possible). The results of that study showed that a reduced amount of presence data in each cell correlated with improved predictions (as assessed by an expert). Drawing from the findings of these studies, datasets with a lower resolution of 30′ were selected for the present study. Consequently, presence data were subjected to simple systematic sampling with a reference grid of 30′ resolution.

The raster files of the variables were clipped with shapefiles from the GADM to create environmental data layers. If a cell containing presence data was lost due to clipping in the shapefile, the feature in the shapefile was reshaped to retain the cell.

## Appendix C

### Evaluator Values

**TABLE A1:** Evaluator values of distribution predictions for *Oxalis latifolia* .

Calibration region	Target region	Evaluator value			Ratio of novel climate areas	Ratio under minimum value	Number of high‐value moving windows without presence data
		AUC	CBI	RWIP			
America	Oceania	0.53	0.35	−0.02	0.00	0.01	0
America, Africa	Oceania	0.86	0.98	0.55	0.00	0.09	0
America, Europe	Oceania	0.89	0.96	0.61	0.00	0.32	0
America, Africa, Europe	Oceania	0.94	0.96	0.84	0.00	0.76	0
America	Africa	0.81	0.93	0.51	0.23	0.15	0
America, Oceania	Africa	0.86	0.97	0.66	0.22	0.04	0
America, Europe	Africa	0.82	0.92	0.51	0.23	0.26	0
America, Oceania, Europe	Africa	0.87	0.93	0.66	0.23	0.06	0
America	Europe	0.89	0.34	0.47	0.06	0.31	0
America, Africa	Europe	0.90	0.66	0.68	0.07	0.35	0
America, Oceania	Europe	0.91	0.82	0.77	0.06	0.32	0
America, Africa, Oceania	Europe	0.91	0.91	0.81	0.06	0.34	0

*Note:* Ratio of novel climate areas: ratio of the number of cells exhibiting negative values in the multivariate environmental similarity surface (MESS) generated by Maxent. Ratio under minimum value: ratio of the number of cells with prediction value below the minimum value of cells containing presence data. Number of high‐value moving windows without presence data: the number of moving windows without any presence data from the highest value moving window (among moving windows with at least one cell).

Abbreviations: AUC, the area under the receiver operating characteristic curve; CBI, continuous Boyce index; RWIP, correlation coefficient with the predictions from the model calibrated in the predicted region.

**TABLE A2:** Evaluator values of distribution predictions for *Digitaria sanguinalis* .

Calibration region	Target region	Evaluator value			Ratio of novel climate areas	Ratio under minimum value	Number of high‐value moving windows without presence data
		AUC	CBI	RWIP			
Europe	Oceania	0.77	0.61	0.37	0.80	0.00	0
Europe, Africa	Oceania	0.95	0.98	0.97	0.00	0.18	0
Europe, North America	Oceania	0.94	1.00	0.90	0.04	0.13	0
Europe, South America	Oceania	0.95	0.99	0.95	0.36	0.36	0
Europe, Africa, North America	Oceania	0.95	0.96	0.94	0.00	0.29	0
Europe, Africa, South America	Oceania	0.95	1.00	0.97	0.00	0.23	0
Europe, North and South America	Oceania	0.92	0.93	0.85	0.00	0.10	1
Europe, Africa, North and South America	Oceania	0.95	0.92	0.95	0.00	0.29	1
Europe	Africa	0.56	0.76	0.17	0.95	0.03	0
Europe, Oceania	Africa	0.83	0.66	0.72	0.63	0.07	3
Europe, North America	Africa	0.85	0.94	0.68	0.44	0.08	0
Europe, South America	Africa	0.84	‐ 0.43	0.59	0.37	0.13	8
Europe, Oceania, North America	Africa	0.84	0.97	0.76	0.40	0.06	0
Europe, Oceania, South America	Africa	0.84	0.03	0.68	0.24	0.05	9
Europe, North and South America	Africa	0.78	0.17	0.46	0.25	0.24	4
Europe, Oceania, North and South America	Africa	0.86	0.47	0.75	0.25	0.17	3
Europe	North America	0.72	0.48	0.57	0.29	0.27	0
Europe, Africa	North America	0.80	0.52	0.76	0.07	0.10	4
Europe, Oceania	North America	0.79	0.31	0.70	0.09	0.08	6
Europe, South America	North America	0.79	0.58	0.69	0.08	0.22	4
Europe, Africa, Oceania	North America	0.80	0.59	0.78	0.06	0.05	3
Europe, Africa, South America	North America	0.80	0.83	0.76	0.06	0.17	1
Europe, Oceania, South America	North America	0.78	0.71	0.70	0.06	0.09	2
Europe, Africa, Oceania, South America	North America	0.79	0.60	0.76	0.06	0.11	4
Europe	South America	0.61	0.43	0.35	0.89	0.00	0
Europe, Africa	South America	0.71	0.65	0.69	0.01	0.00	3
Europe, Oceania	South America	0.72	0.39	0.76	0.50	0.00	7
Europe, North America	South America	0.72	0.92	0.78	0.16	0.01	0
Europe, Africa, Oceania	South America	0.71	0.51	0.72	0.01	0.00	5
Europe, Africa, North America	South America	0.70	0.66	0.72	0.01	0.00	4
Europe, Oceania, North America	South America	0.73	0.70	0.82	0.15	0.01	4
Europa, Africa, Oceania, North America	South America	0.71	0.66	0.75	0.01	0.00	4

*Note:* Ratio of novel climate areas: ratio of the number of cells exhibiting negative values in the multivariate environmental similarity surface (MESS) generated by Maxent. Ratio under minimum value: ratio of the number of cells with prediction value below the minimum value of cells containing presence data. Number of high‐value moving windows without presence data: the number of moving windows without any presence data from the highest value moving window (among moving windows with at least one cell).

Abbreviations: AUC, the area under the receiver operating characteristic curve; CBI, continuous Boyce index; RWIP, correlation coefficient with the predictions from the model calibrated in the predicted region.

**TABLE A3:** Evaluator values of distribution predictions for *Amaranthus retroflexus* .

Calibration region	Target region	Evaluator value			Ratio of novel climate areas	Ratio under minimum value	Number of high‐value moving windows without presence data
		AUC	CBI	RWIP			
North America	Oceania	0.92	0.71	0.87	0.03	0.42	4
North America, East Asia	Oceania	0.92	0.92	0.83	0.03	0.45	1
North America, Europe	Oceania	0.92	0.96	0.90	0.04	0.00	0
North America, East Asia, Europe	Oceania	0.92	0.80	0.88	0.03	0.10	3
North America	East Asia	0.79	0.84	0.85	0.02	0.10	1
North America, Oceania	East Asia	0.80	0.93	0.89	0.02	0.11	0
North America, Europe	East Asia	0.76	0.37	0.74	0.02	0.09	3
North America, Oceania, Europe	East Asia	0.76	0.42	0.76	0.02	0.09	3
North America	Europe	0.81	0.98	0.92	0.00	0.04	0
North America, Oceania	Europe	0.80	0.95	0.90	0.00	0.04	0
North America, East Asia	Europe	0.81	0.98	0.90	0.00	0.04	0
North America, Oceania, East Asia	Europe	0.80	0.93	0.89	0.00	0.04	0

*Note:* Ratio of novel climate areas: ratio of the number of cells exhibiting negative values in the multivariate environmental similarity surface (MESS) generated by Maxent. Ratio under minimum value: ratio of the number of cells with prediction value below the minimum value of cells containing presence data. Number of high‐value moving windows without presence data: the number of moving windows without any presence data from the highest value moving window (among moving windows with at least one cell).

Abbreviations: AUC, the area under the receiver operating characteristic curve; CBI, continuous Boyce index; RWIP, correlation coefficient with the predictions from the model calibrated in the predicted region.

**TABLE A4:** Evaluator values of random holdout cross‐validation for the three species.

Species	Regions of training data	Evaluator value	
		AUC	CBI
*Oxalis latifolia*	America	0.87	0.96
	America, Africa	0.87	0.96
	America, Europe	0.87	0.98
	America, Oceania	0.86	0.94
	America, Africa, Europe	0.88	0.98
	America, Africa, Oceania	0.86	0.97
	America, Europe, Oceania	0.87	0.97
*Digitaria sanguinalis*	Europe	0.82	0.97
	Europe, Africa	0.89	0.98
	Europe, Oceania	0.85	0.97
	Europe, North America	0.81	0.99
	Europe, South America	0.87	0.98
	Europe, Africa, Oceania	0.88	0.99
	Europe, Africa, North America	0.84	0.99
	Europe, Africa, South America	0.88	0.99
	Europe, Oceania, North America	0.81	0.99
	Europe, Oceania, South America	0.87	0.99
	Europe, North and South America	0.83	1.00
	Europe, Africa, Oceania, North America	0.83	0.99
	Europe, Africa, Oceania, South America	0.88	0.99
	Europe, Africa, North and South America	0.84	0.99
	Europe, Oceania, North and South America	0.82	0.99
*Amaranthus retroflexus*	North America	0.85	0.97
	North America, East Asia	0.83	0.98
	North America, Europe	0.80	0.99
	North America, Oceania	0.86	0.96
	North America, East Asia, Europe	0.80	1.00
	North America, East Asia, Oceania	0.84	0.98
	North America, Europe, Oceania	0.81	0.98

*Note:* Evaluator value: average value of 4‐fold cross‐validation.

Abbreviations: AUC, the area under the receiver operating characteristic curve; CBI, continuous Boyce index.

## Appendix D

### Relationship Between Ratio of Novel Climate Areas and Evaluator Values

The discrepancies in model performance between predictions on the random holdout test dataset and those in the target region may arise when predictor variable values in the target region fall outside the range observed during model training. The distribution data of a species alone provide no information on how the species may respond to novel environments (Fitzpatrick and Hargrove 2009). In such cases, Maxent treats variables outside the training range as being at the limits of the training range (“clamping”). In this study, the effect of novel climate on the distribution predictions for the three species was examined.

#### Methods

Whether novel climate affects the predictive performance was inferred from the correlation coefficient between the ratios of the novel climate areas and the evaluator values in the target region. When predicting species distribution, Maxent generates an accompanying map displaying the multivariate environmental similarity surface (MESS) value for each grid cell (Elith et al. 2010). In MESS, a cell with a negative value indicates the presence of at least one predictor variable with a value that falls outside the range of the training data. The ratio of the number of cells with negative values to the total number of cells in the target region was calculated to obtain the ratio of novel climate areas.

#### Results

The ratios of novel climate areas in the target regions for *O. latifolia*, *D. sanguinalis*, *A. retroflexus* were 0.00–0.23, 0.00–0.95, and 0.00–0.04 respectively (Tables A1, A2, A3 in Appendix C). The ratios for *O. latifolia* varied depending on the target region. The variation in the ratios for *D. sanguinalis* was more significantly affected by the calibration regions.

In the distribution predictions of *O. latifolia*, correlation coefficients between the ratio of the novel climate areas in the target region and the evaluator value of predictions were positive (*r* = 0.04 [AUC], *r* = 0.32 [CBI], *r* = 0.08 [RWIP]), however, no correlation was observed (*p* = 0.90 [AUC], *p* = 0.31 [CBI], *p* = 0.80 [RWIP]) (Figure A1). In the distribution predictions of *D. sanguinalis*, a strong negative correlation was observed between the abovementioned parameters when the RWIP was used (*r* = −0.74, *p* = 1.1 × 10^−6^), a negative correlation was observed when the AUC was used (*r* = −0.44, *p* = 0.011), and no correlation was observed when the CBI was used (*r* = −0.20, *p* = 0.27) (Figure A2). In the distribution predictions of *A. retroflexus*, a positive correlation was observed when the AUC was used (*r* = 0.65, *p* = 0.023), and no correlation was observed when the CBI or RWIP was used (*r* = −0.27 and *r* = −0.29, *p* = 0.40 and *p* = 0.37, respectively) (Figure A3).

#### Conclusion

The correlations described above did not suggest that including novel climate areas had a negative effect on the prediction accuracy for the distribution of the two species aside from *D. sanguinalis*.

## Appendix E

### Paired Predictions from the Same Models in Different Regions

**TABLE A5:** Evaluator values of paired predictions from the same models in different regions.

Species	Calibration region	Paired target regions		Evaluator values of distribution prediction in the target region							
				Target region	AUC	CBI	RWIP	Target region	AUC	CBI	RWIP
*Oxalis latifolia*	America	Oceania	Africa	Oceania	0.53	0.35	−0.02	Africa	0.81	0.93	0.51
	America	Oceania	Europe	Oceania	0.53	0.35	−0.02	Europe	0.89	0.34	0.47
	America	Africa	Europe	Africa	0.81	0.93	0.51	Europe	0.89	0.34	0.47
	America, Africa	Oceania	Europe	Oceania	0.86	0.98	0.55	Europe	0.90	0.66	0.68
	America, Oceania	Africa	Europe	Africa	0.86	0.97	0.66	Europe	0.91	0.82	0.77
	America, Europe	Oceania	Africa	Oceania	0.89	0.96	0.61	Africa	0.82	0.92	0.51
*Digitaria sanguinalis*	Europe	Oceania	Africa	Oceania	0.77	0.61	0.37	Africa	0.56	0.76	0.17
	Europe	Oceania	North America	Oceania	0.77	0.61	0.37	North America	0.72	0.48	0.57
	Europe	Oceania	South America	Oceania	0.77	0.61	0.37	South America	0.61	0.43	0.35
	Europe	Africa	North America	Africa	0.56	0.76	0.17	North America	0.72	0.48	0.57
	Europe	Africa	South America	Africa	0.56	0.76	0.17	South America	0.61	0.43	0.35
	Europe	North America	South America	North America	0.72	0.48	0.57	South America	0.61	0.43	0.35
	Europe, Africa	Oceania	North America	Oceania	0.95	0.98	0.97	North America	0.80	0.52	0.76
	Europe, Africa	Oceania	South America	Oceania	0.95	0.98	0.97	South America	0.71	0.65	0.69
	Europe, Africa	North America	South America	North America	0.80	0.52	0.76	South America	0.71	0.65	0.69
	Europe, Oceania	Africa	North America	Africa	0.83	0.66	0.72	North America	0.79	0.31	0.70
	Europe, Oceania	Africa	South America	Africa	0.83	0.66	0.72	South America	0.72	0.39	0.76
	Europe, Oceania	North America	South America	North America	0.79	0.31	0.70	South America	0.72	0.39	0.76
	Europe, North America	Oceania	Africa	Oceania	0.94	1.00	0.90	Africa	0.85	0.94	0.68
	Europe, North America	Oceania	South America	Oceania	0.94	1.00	0.90	South America	0.72	0.92	0.78
	Europe, North America	Africa	South America	Africa	0.85	0.94	0.68	South America	0.72	0.92	0.78
	Europe, South America	Oceania	Africa	Oceania	0.95	0.99	0.95	Africa	0.84	−0.43	0.59
	Europe, South America	Oceania	North America	Oceania	0.95	0.99	0.95	North America	0.79	0.58	0.69
	Europe, South America	Africa	North America	Africa	0.84	−0.43	0.59	North America	0.79	0.58	0.69
	Europe, Africa, Oceania	North America	South America	North America	0.80	0.59	0.78	South America	0.71	0.51	0.72
	Europe, Africa, North America	Oceania	South America	Oceania	0.95	0.96	0.94	South America	0.70	0.66	0.72
	Europe, Africa, South America	Oceania	North America	Oceania	0.95	1.00	0.97	North America	0.80	0.83	0.76
	Europe, Oceania, North America	Africa	South America	Africa	0.84	0.97	0.76	South America	0.73	0.70	0.82
	Europe, Oceania, South America	Africa	North America	Africa	0.84	0.03	0.68	North America	0.78	0.71	0.70
	Europe, North and South America	Oceania	Africa	Oceania	0.92	0.93	0.85	Africa	0.78	0.17	0.46
*Amaranthus retroflexus*	North America	Oceania	East Asia	Oceania	0.92	0.71	0.87	East Asia	0.79	0.84	0.85
	North America	Oceania	Europe	Oceania	0.92	0.71	0.87	Europe	0.81	0.98	0.92
	North America	East Asia	Europe	East Asia	0.79	0.84	0.85	Europe	0.81	0.98	0.92
	North America, East Asia	Oceania	Europe	Oceania	0.92	0.92	0.83	Europe	0.81	0.98	0.90
	North America, Oceania	East Asia	Europe	East Asia	0.80	0.93	0.89	Europe	0.80	0.95	0.90
	North America, Europe	Oceania	East Asia	Oceania	0.92	0.96	0.90	East Asia	0.76	0.37	0.74

Abbreviations: AUC, the area under the receiver operating characteristic curve; CBI, continuous Boyce index; RWIP, correlation coefficient with the predictions from the model calibrated in the predicted region.
