# Correction to “Assessing the Transferability of Species Distribution Models: A Cross‐Continental Evaluation”

**DOI:** 10.1002/ece3.73828

**Published:** 2026-06-17

Matsui, T. 2026. “Assessing the Transferability of Species Distribution Models: A Cross‐Continental Evaluation.” *Ecology and Evolution* 16, no. 5: e73534. https://doi.org/10.1002/ece3.73534.

In the sixth paragraph of Section 2.2, “three different calibration datasets” is corrected to “four different calibration datasets.” This number is the same as the number of combinations in parentheses in the “Calibration regions” column of Table 2.

The correct sentence should read as follows:

“Therefore, four different calibration datasets were used for one target region in the case of *O. latifolia* and *A. retroflexus*, whereas eight different calibration datasets were employed for one target region in the case of *D. sanguinalis*.”

The Data Availability Statement section was incorrect. It should read as follows:

**Data Availability Statement**

The data and code that support the findings of this study are openly available in Dryad at https://doi.org/10.5061/dryad.m37pvmdgc, and in Zenodo at https://doi.org/10.5281/zenodo.19970795.

We apologize for these errors.

