# Supplementary material for: Assessing the Transferability of Species Distribution Models: A Cross‐Continental Evaluation
Source: Ecol Evol. 2026 Apr 29;16(5):e73534. doi: 10.1002/ece3.73534 (PMC13128341; doi:10.1002/ece3.73534)
Supplement: Supplementary file 1 — Figure S1: Distribution predictions for Oxalis latifolia . Figure S2: Distribution predictions for Digitaria sanguinalis . Figure S3: Distribution predictions for Amaranthus retroflexus . [file ECE3-16-e73534-s001.pdf]

Supporting information

Assessing the transferability of species distribution models

Figure S1. Distribution predictions for *Oxalis latifolia*

Figure S2. Distribution predictions for *Digitaria sanguinalis*

Figure S3. Distribution predictions for *Amaranthus retroflexus*

Figure S1. Distribution predictions for *Oxalis latifolia*

Target region: Oceania

[1] Training data: America [2] Training data: America, Africa [3] Training data: America, Europe [4] Training data: America, Africa, Europe

Target region: Africa

[5] Training data: America [6] Training data: America, Oceania [7] Training data: America, Europe [8] Training data: America, Oceania, Europe

Target region: Europe

[9] Training data: America [10] Training data: America, Africa [11] Training data: America, Oceania [12] Training data: America, Africa, Oceania

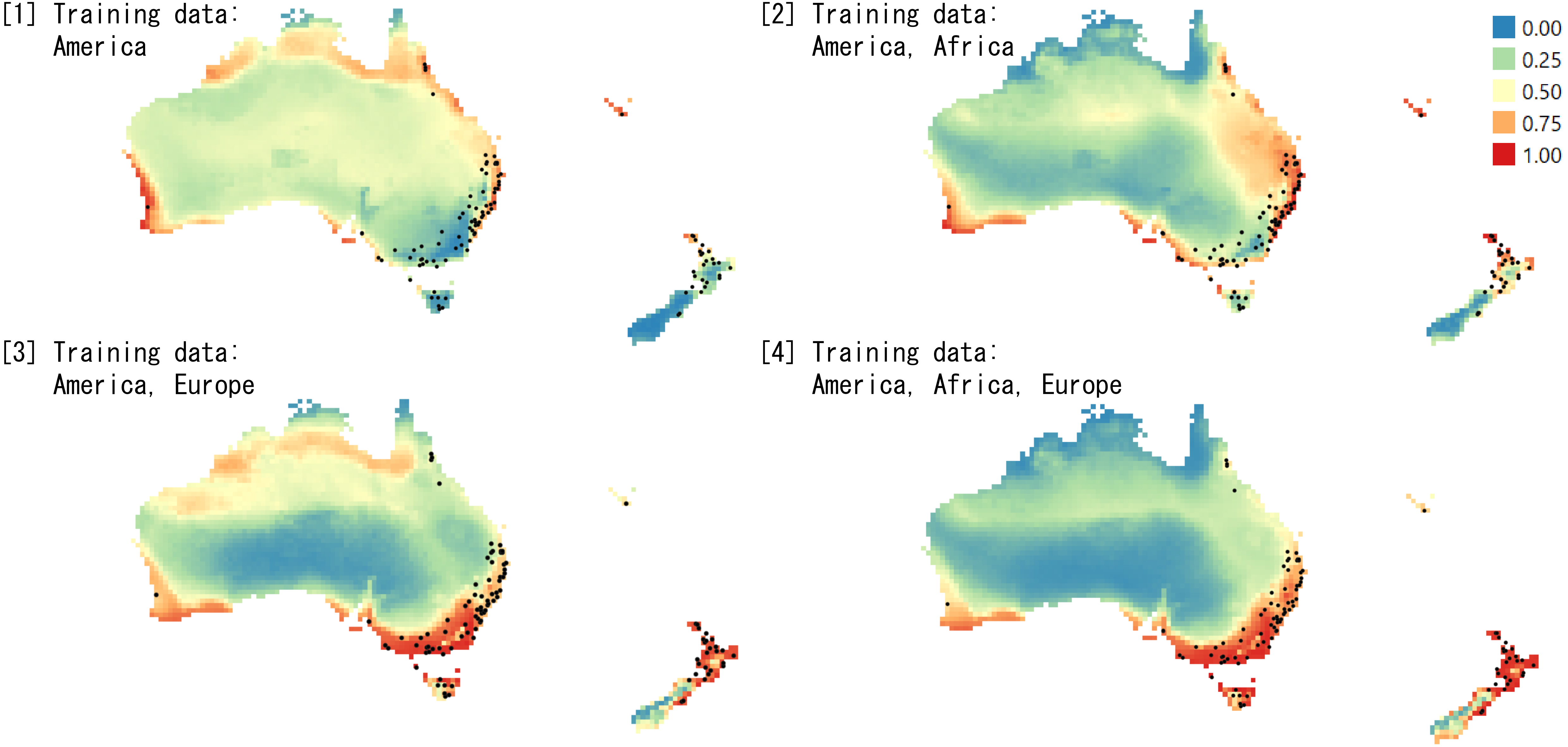

Figure S1. Distribution predictions for *Oxalis latifolia* (target region: Oceania)

Maps displaying the Maxent output values; the legend is located in the top right of the figure.

The black dots on the maps represent distribution points. (To make the distribution easier to visualize on the maps, the data were subjected to simple systematic sampling with a reference grid of 30′ resolution.)

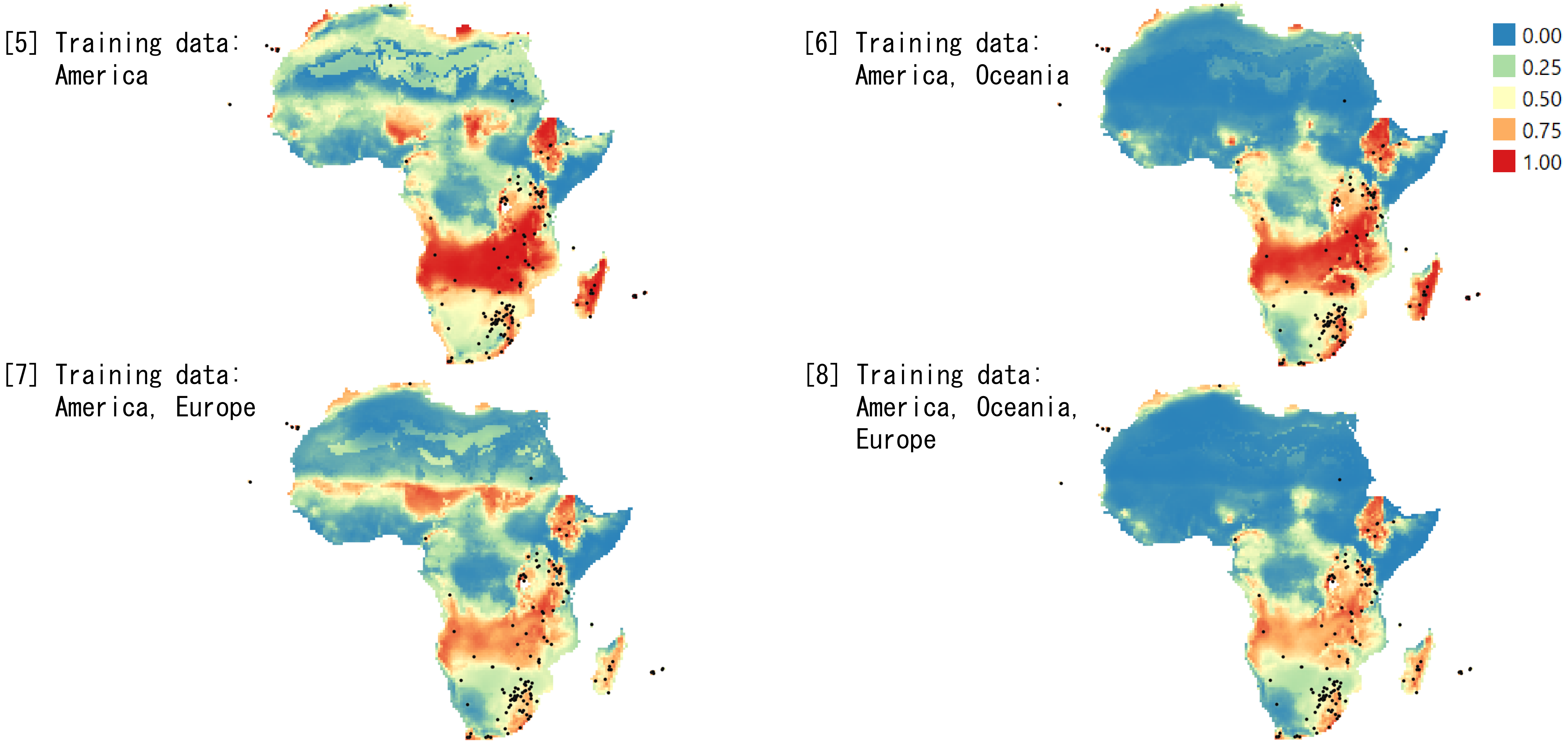

Figure S1. Distribution predictions for *Oxalis latifolia* (target region: Africa)

Maps displaying the Maxent output values; the legend is located in the top right of the figure.
The black dots on the maps represent distribution points. (To make the distribution easier to visualize on the maps, the data were subjected to simple systematic sampling with a reference grid of 30′ resolution.)

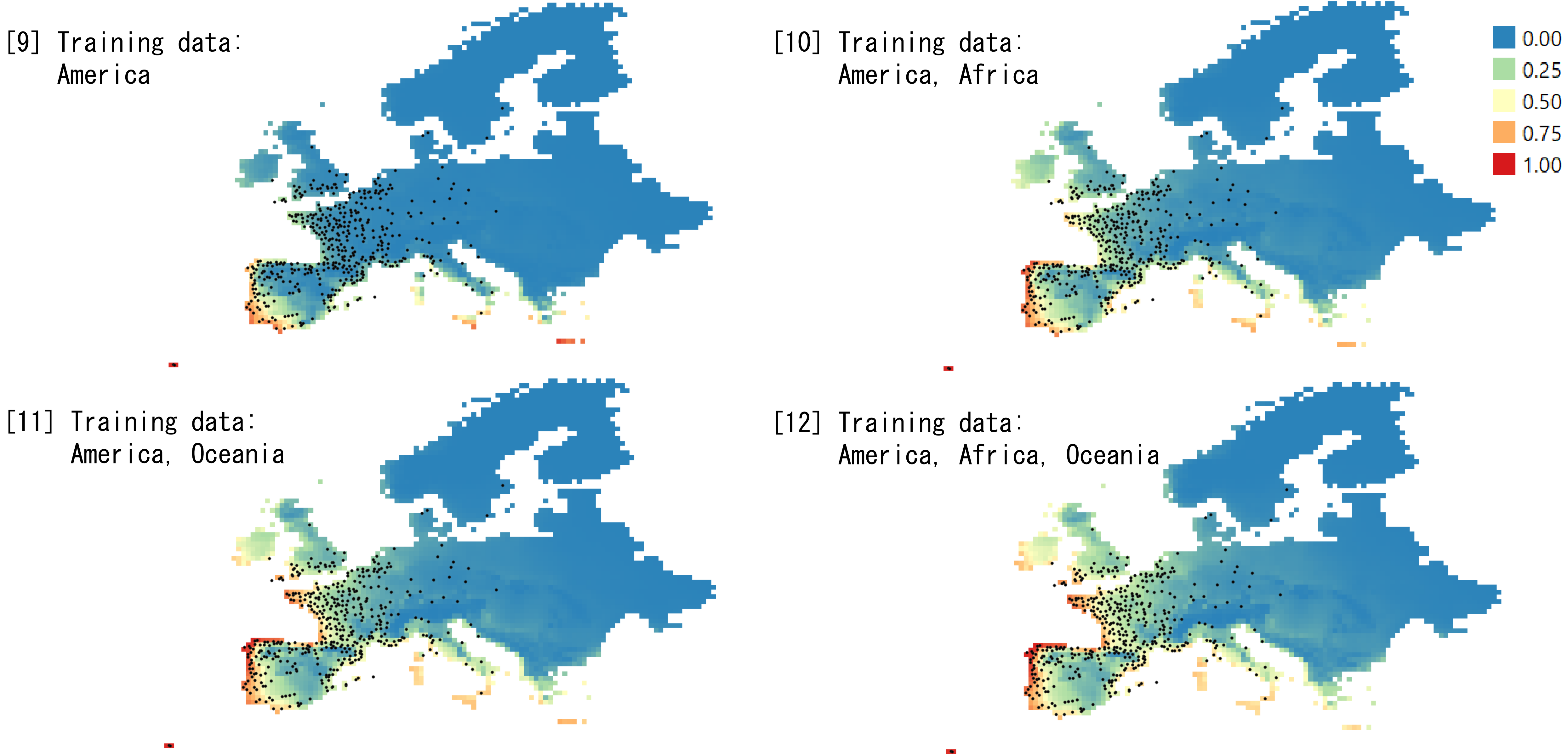

Figure S1. Distribution predictions for *Oxalis latifolia* (target region: Europe)

Maps displaying the Maxent output values; the legend is located in the top right of the figure.

The black dots on the maps represent distribution points. (To make the distribution easier to visualize on the maps, the data were subjected to simple systematic sampling with a reference grid of 30′ resolution.)

Figure S2. Distribution predictions for *Digitaria sanguinalis*

Target region: Oceania

[1] Training data: Europe [2] Training data: Europe, Africa [3] Training data: Europe, North America [4] Training data: Europe, South America
[5] Training data: Europe, Africa, North America [6] Training data: Europe, Africa, South America [7] Training data: Europe, North and South America
[8] Training data: Europe, Africa, North and South America

Target region: Africa

[9] Training data: Europe [10] Training data: Europe, Oceania [11] Training data: Europe, North America [12] Training data: Europe, South America
[13] Training data: Europe, Oceania, North America [14] Training data: Europe, Oceania, South America
[15] Training data: Europe, North and South America [16] Training data: Europe, Oceania, North and South America

Target region: North America

[17] Training data: Europe [18] Training data: Europe, Africa [19] Training data: Europe, Oceania [20] Training data: Europe, South America
[21] Training data: Europe, Africa, Oceania [22] Training data: Europe, Africa, South America [23] Training data: Europe, Oceania, South America
[24] Training data: Europe, Africa, Oceania, South America

Target region: South America

[25] Training data: Europe [26] Training data: Europe, Africa [27] Training data: Europe, Oceania [28] Training data: Europe, North America
[29] Training data: Europe, Africa, Oceania [30] Training data: Europe, Africa, North America [31] Training data: Europe, Oceania, North America
[32] Training data: Europe, Africa, Oceania, North America

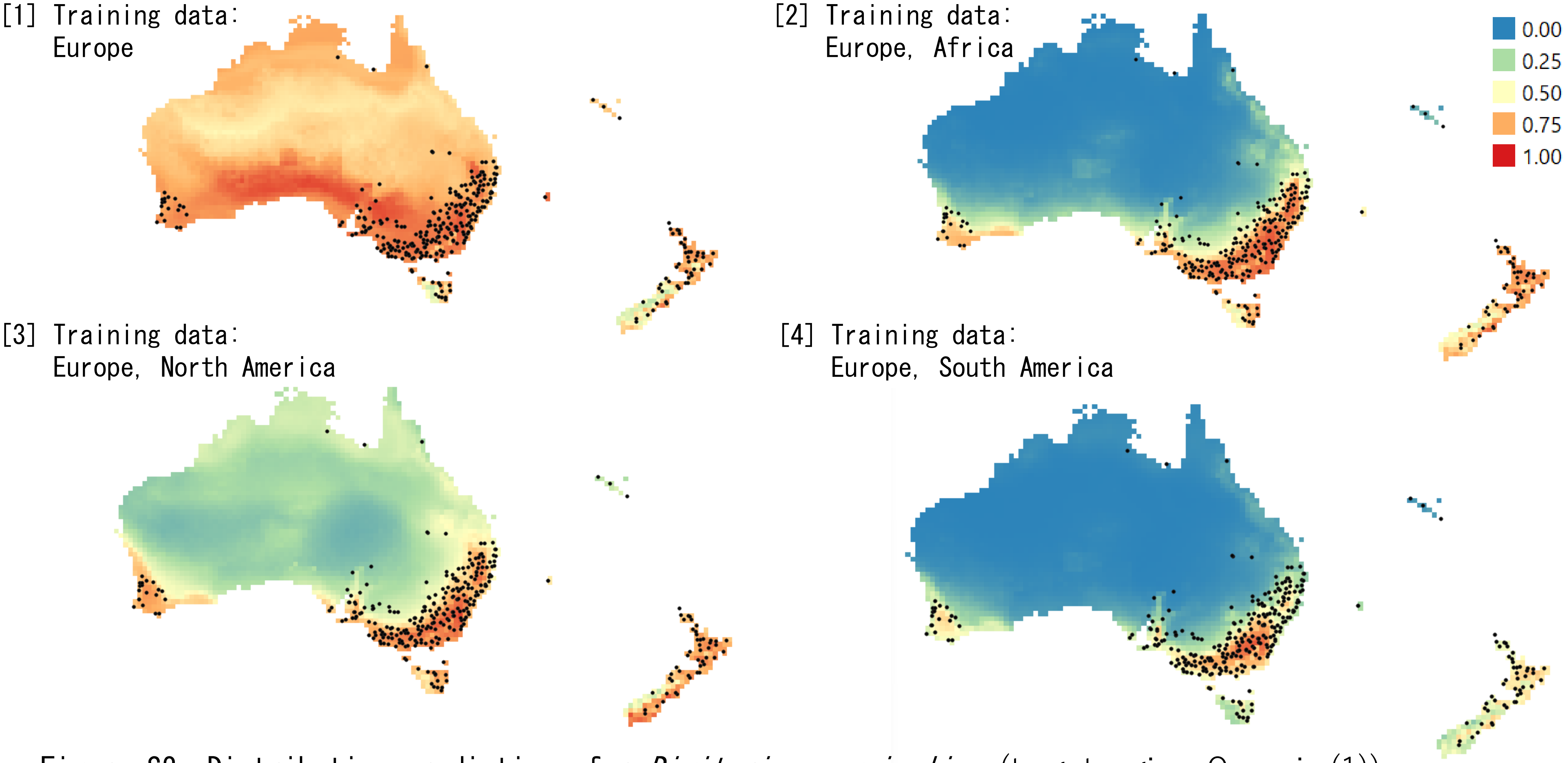

Figure S2. Distribution predictions for *Digitaria sanguinalis* (target region: Oceania (1))

Maps displaying the Maxent output values; the legend is located in the top right of the figure.
The black dots on the maps represent distribution points. (To make the distribution easier to visualize on the maps, the data were subjected to simple systematic sampling with a reference grid of 30′ resolution.)

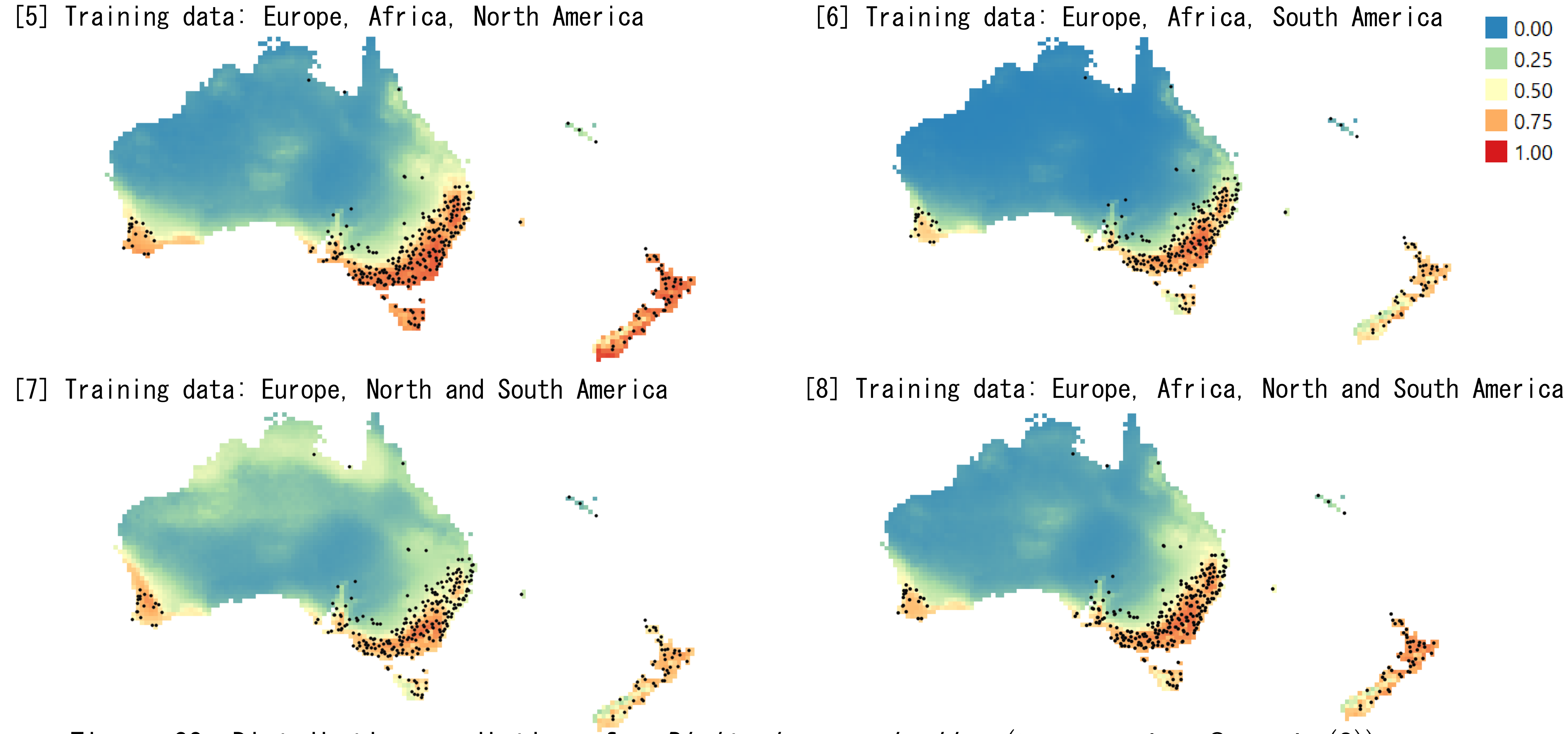

Figure S2. Distribution predictions for *Digitaria sanguinalis* (target region: Oceania (2))

Maps displaying the Maxent output values; the legend is located in the top right of the figure.
The black dots on the maps represent distribution points. (To make the distribution easier to visualize on the maps, the data were subjected to simple systematic sampling with a reference grid of 30′ resolution.)

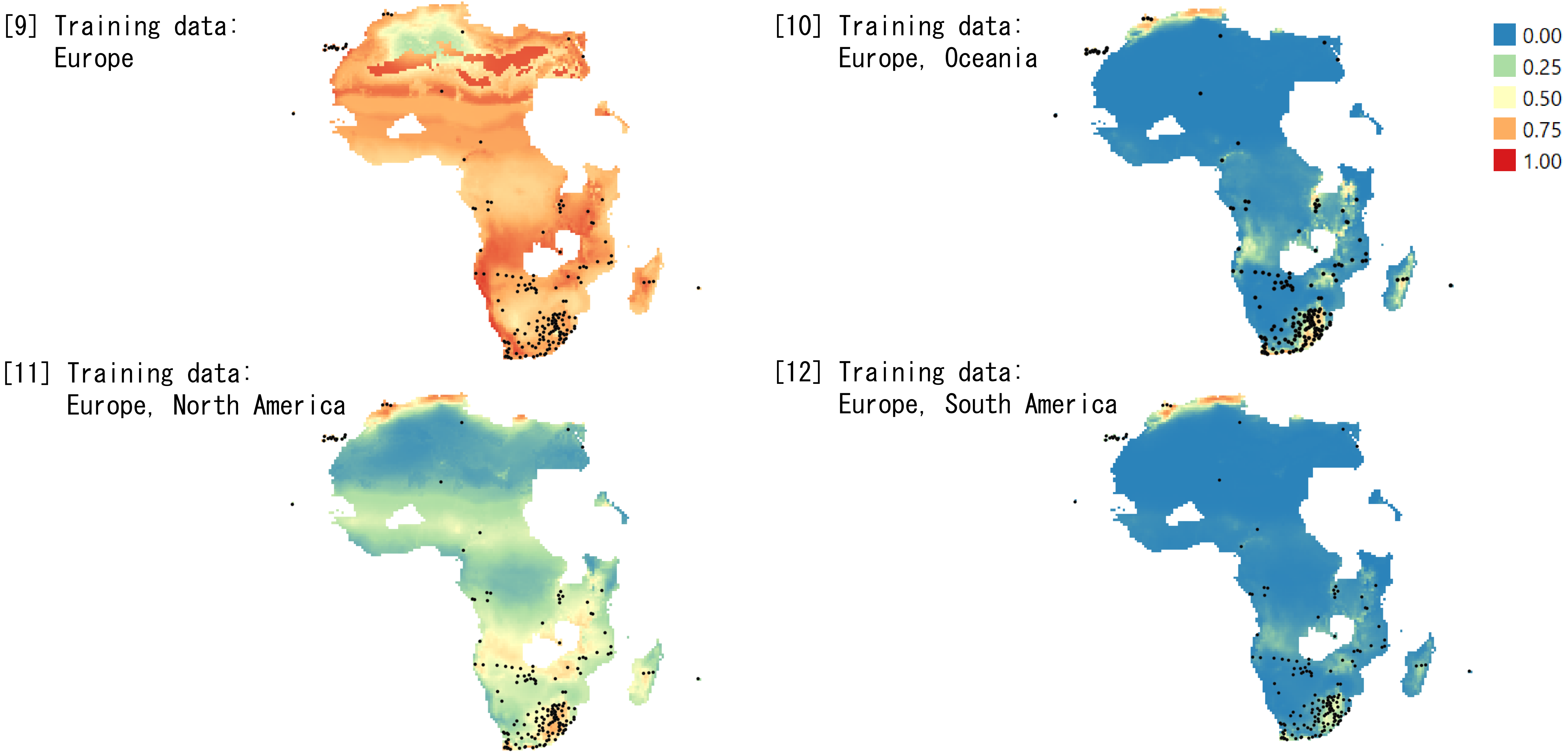

Figure S2. Distribution predictions for *Digitaria sanguinalis* (target region: Africa (1))

Maps displaying the Maxent output values; the legend is located in the top right of the figure.
The black dots on the maps represent distribution points. (To make the distribution easier to visualize on the maps, the data were subjected to simple systematic sampling with a reference grid of 30′ resolution.)

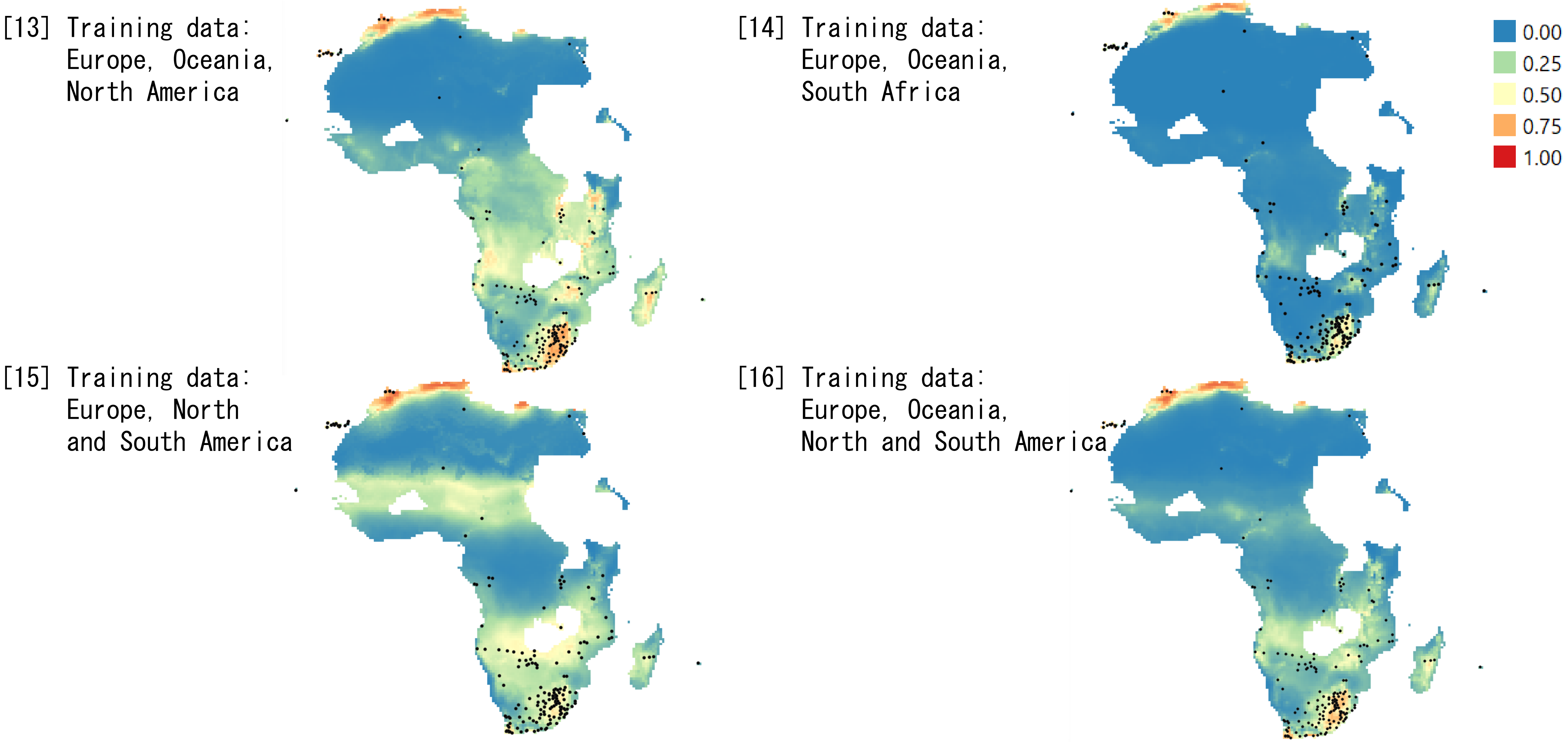

Figure S2. Distribution predictions for *Digitaria sanguinalis* (target region: Africa (2))

Maps displaying the Maxent output values; the legend is located in the top right of the figure.
The black dots on the maps represent distribution points. (To make the distribution easier to visualize on the maps, the data were subjected to simple systematic sampling with a reference grid of 30′ resolution.)

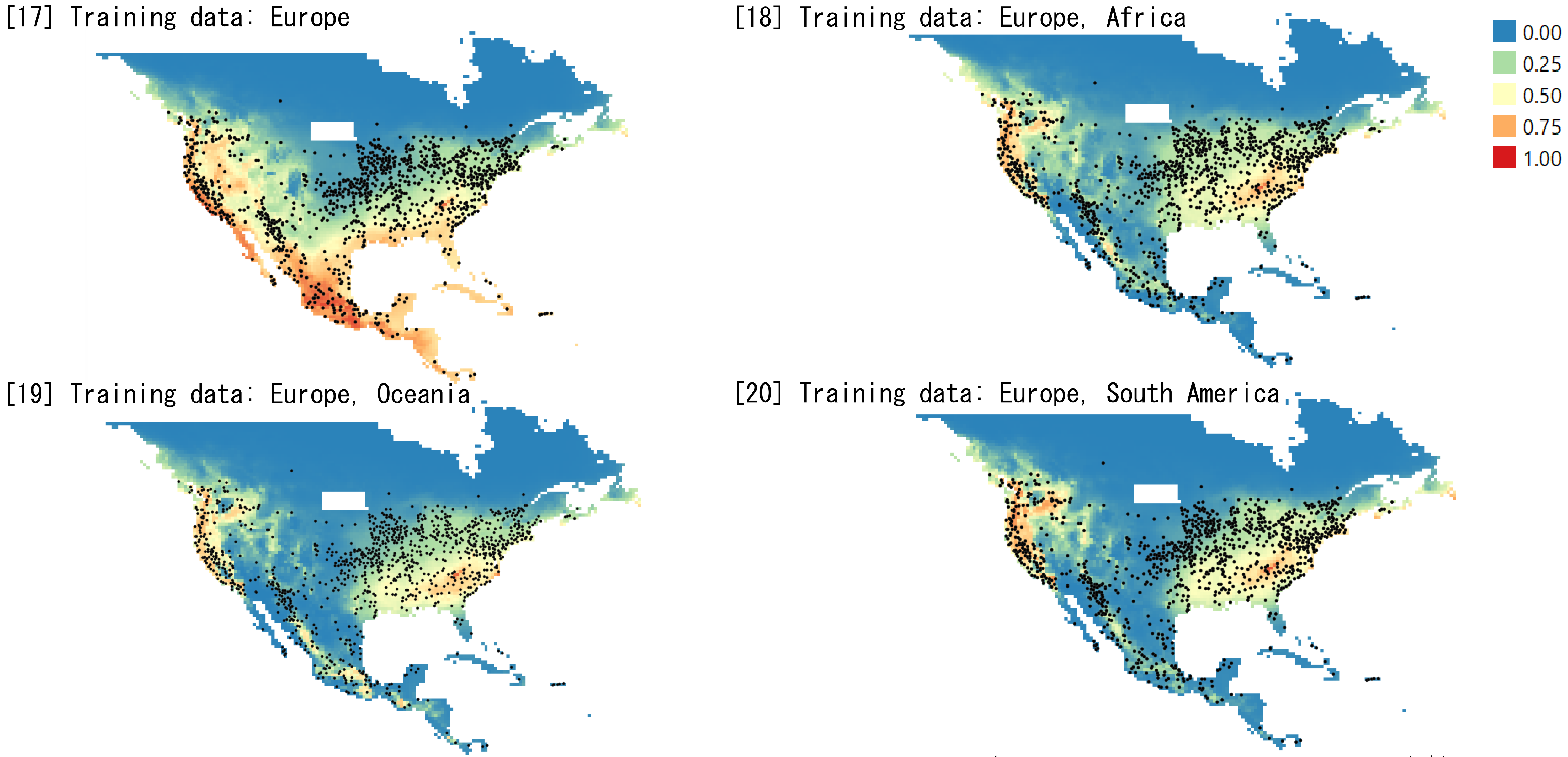

Figure S2. Distribution predictions for *Digitaria sanguinalis* (target region: North America (1))

Maps displaying the Maxent output values; the legend is located in the top right of the figure.
The black dots on the maps represent distribution points. (To make the distribution easier to visualize on the maps, the data were subjected to simple systematic sampling with a reference grid of 30′ resolution.)

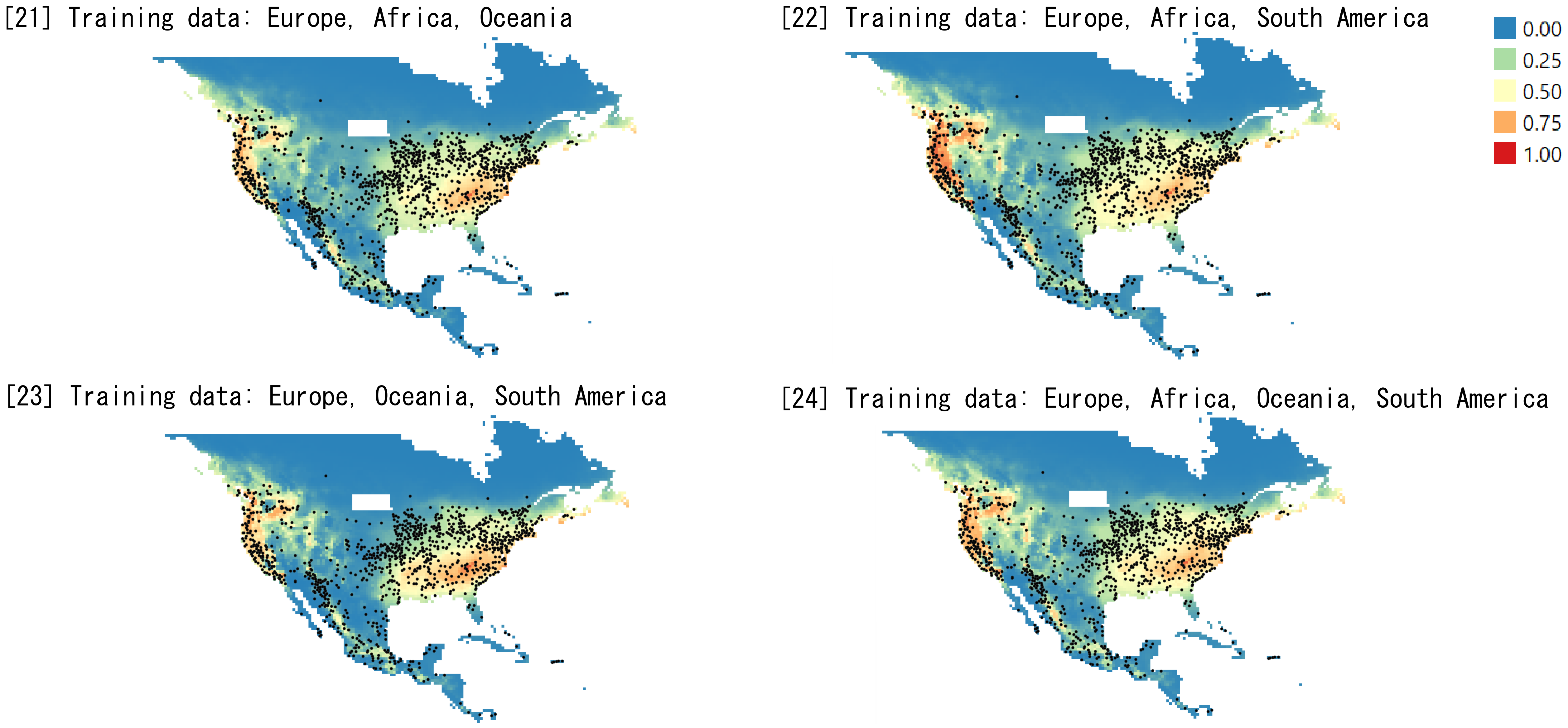

Figure S2. Distribution predictions for *Digitaria sanguinalis* (target region: North America (2))

Maps displaying the Maxent output values; the legend is located in the top right of the figure.
The black dots on the maps represent distribution points. (To make the distribution easier to visualize on the maps, the data were subjected to simple systematic sampling with a reference grid of 30′ resolution.)

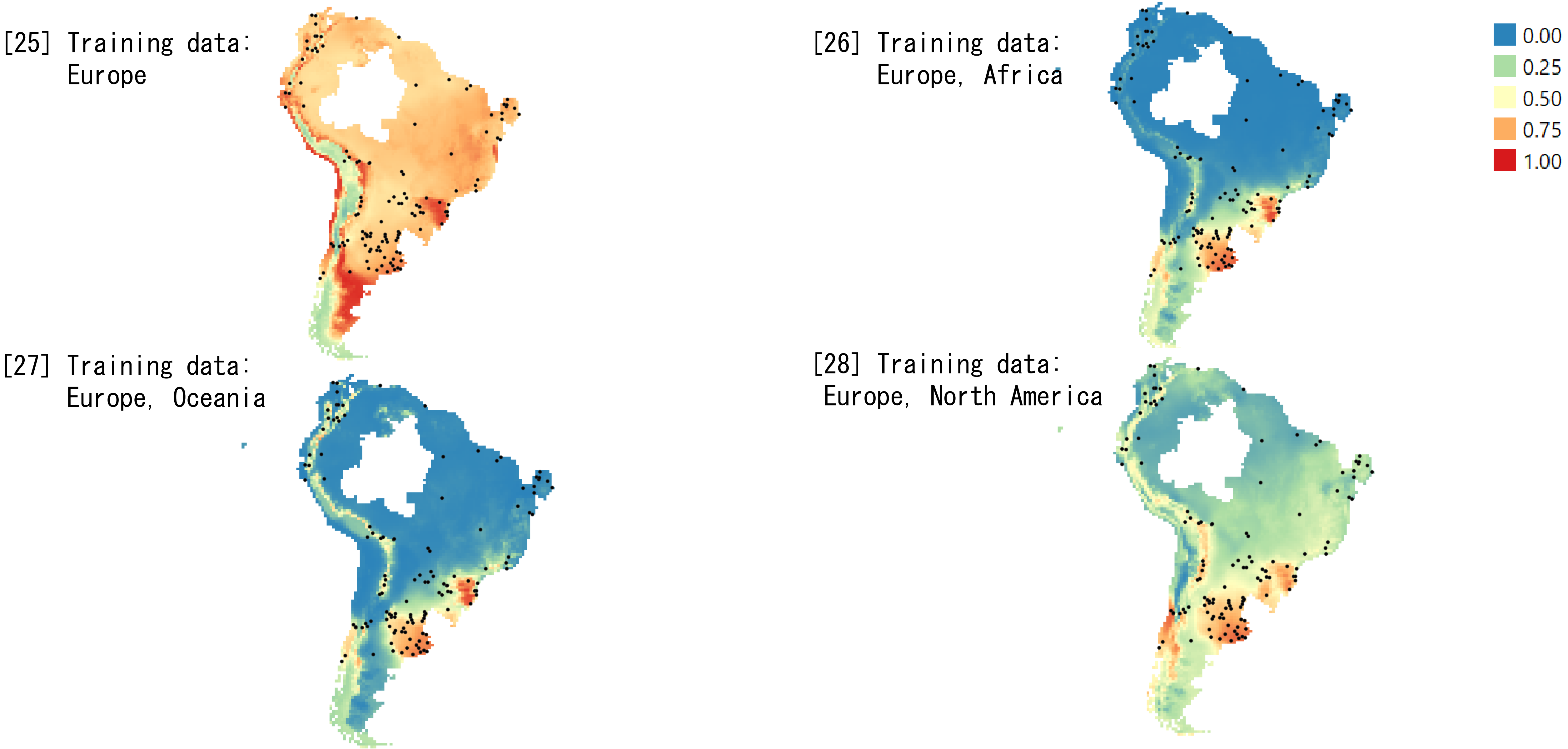

Figure S2. Distribution predictions for *Digitaria sanguinalis* (target region: South America (1))

Maps displaying the Maxent output values; the legend is located in the top right of the figure.
The black dots on the maps represent distribution points. (To make the distribution easier to visualize on the maps, the data were subjected to simple systematic sampling with a reference grid of 30′ resolution.)

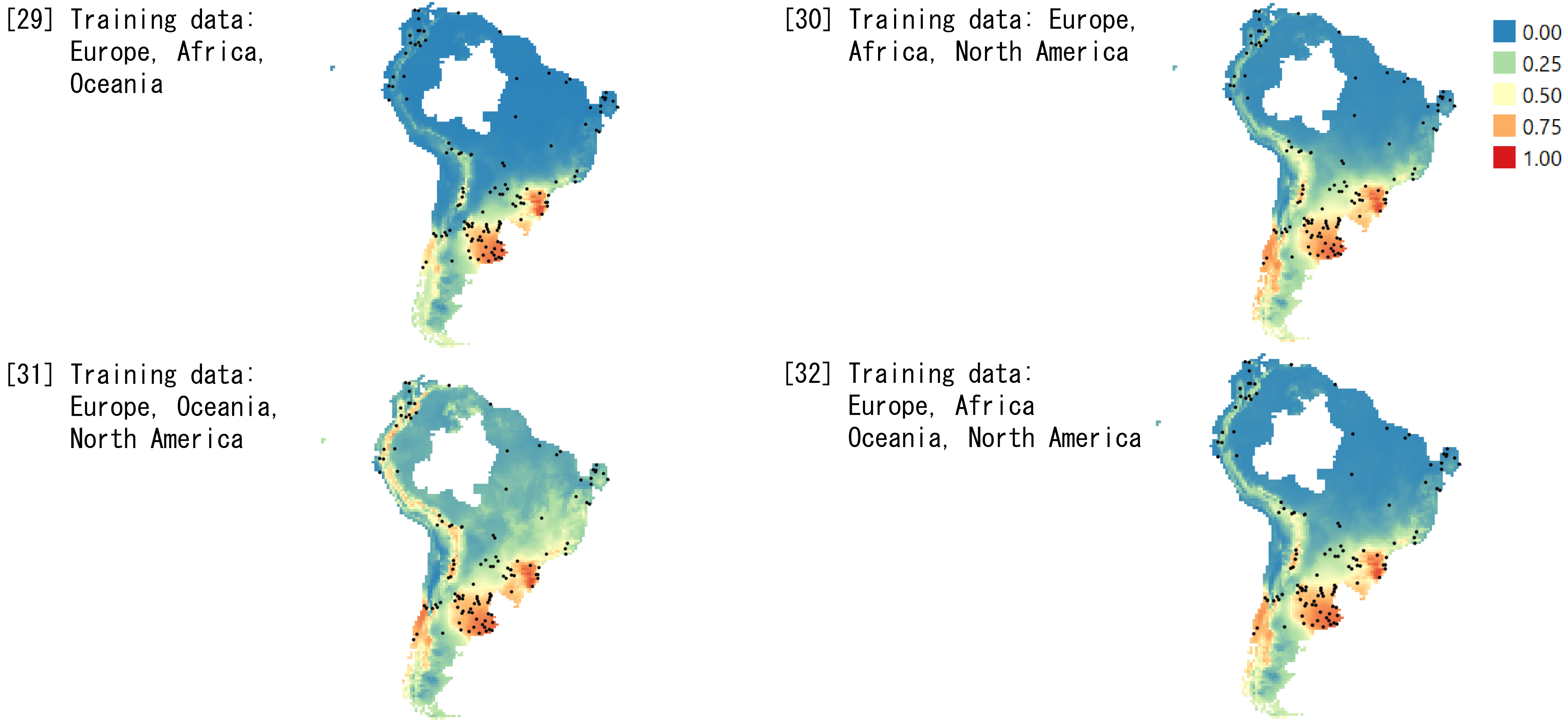

Figure S2. Distribution predictions for *Digitaria sanguinalis* (target region: South America (2))

Maps displaying the Maxent output values; the legend is located in the top right of the figure.
The black dots on the maps represent distribution points. (To make the distribution easier to visualize on the maps, the data were subjected to simple systematic sampling with a reference grid of 30′ resolution.)

Figure S3. Distribution predictions for *Amaranthus retroflexus*

Target region: Oceania

[1] Training data: North America [2] Training data: North America, East Asia [3] Training data: North America, Europe
[4] Training data: North America, East Asia, Europe

Target region: East Asia

[5] Training data: North America [6] Training data: North America, Oceania [7] Training data: North America, Europe
[8] Training data: North America, Oceania, Europe

Target region: Europe

[9] Training data: North America [10] Training data: North America, Oceania [11] Training data: North America, East Asia
[12] Training data: North America, Oceania, East Asia

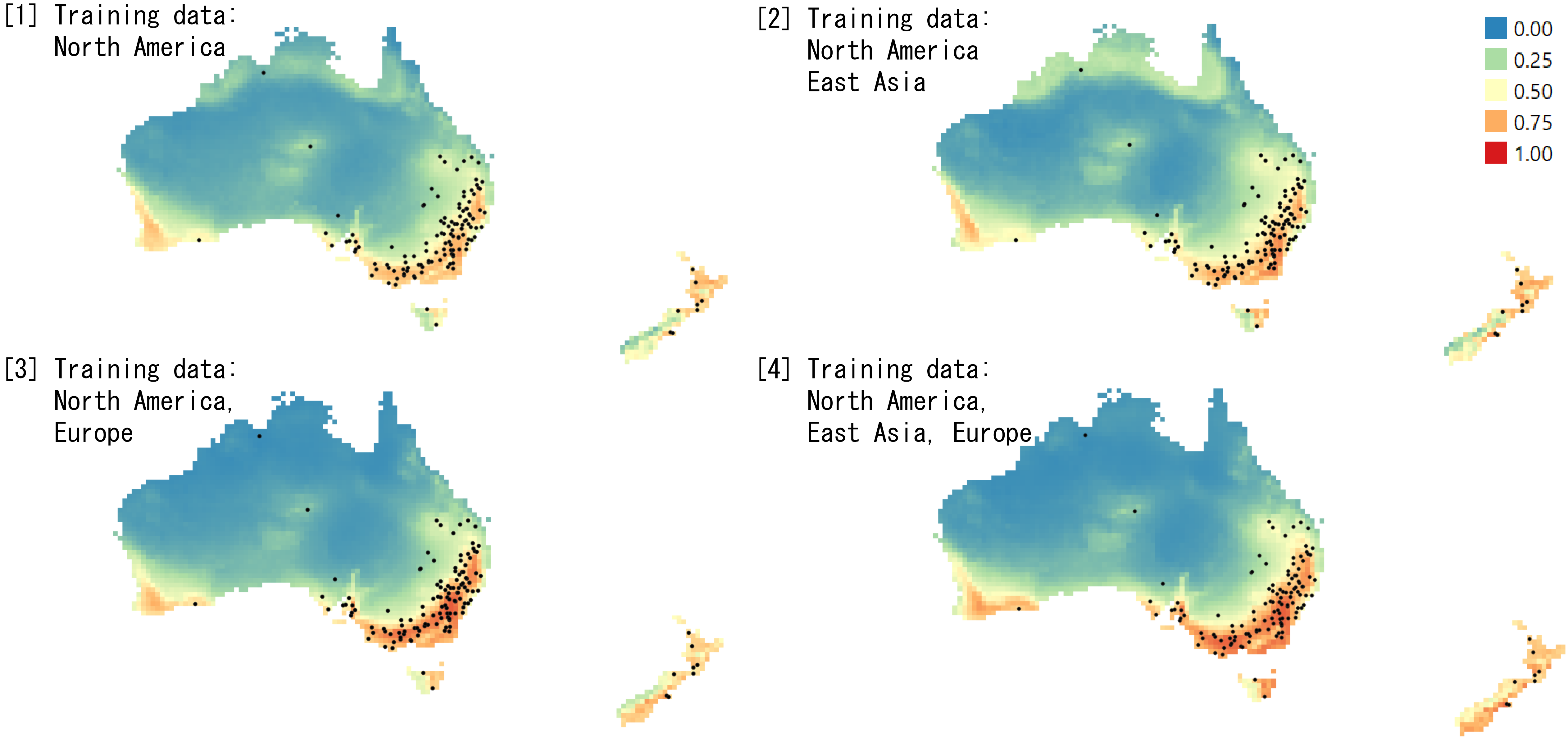

Figure S3. Distribution predictions for *Amaranthus retroflexus* (target region: Oceania)

Maps displaying the Maxent output values; the legend is located in the top right of the figure.

The black dots on the maps represent distribution points. (To make the distribution easier to visualize on the maps, the data were subjected to simple systematic sampling with a reference grid of 30′ resolution.)

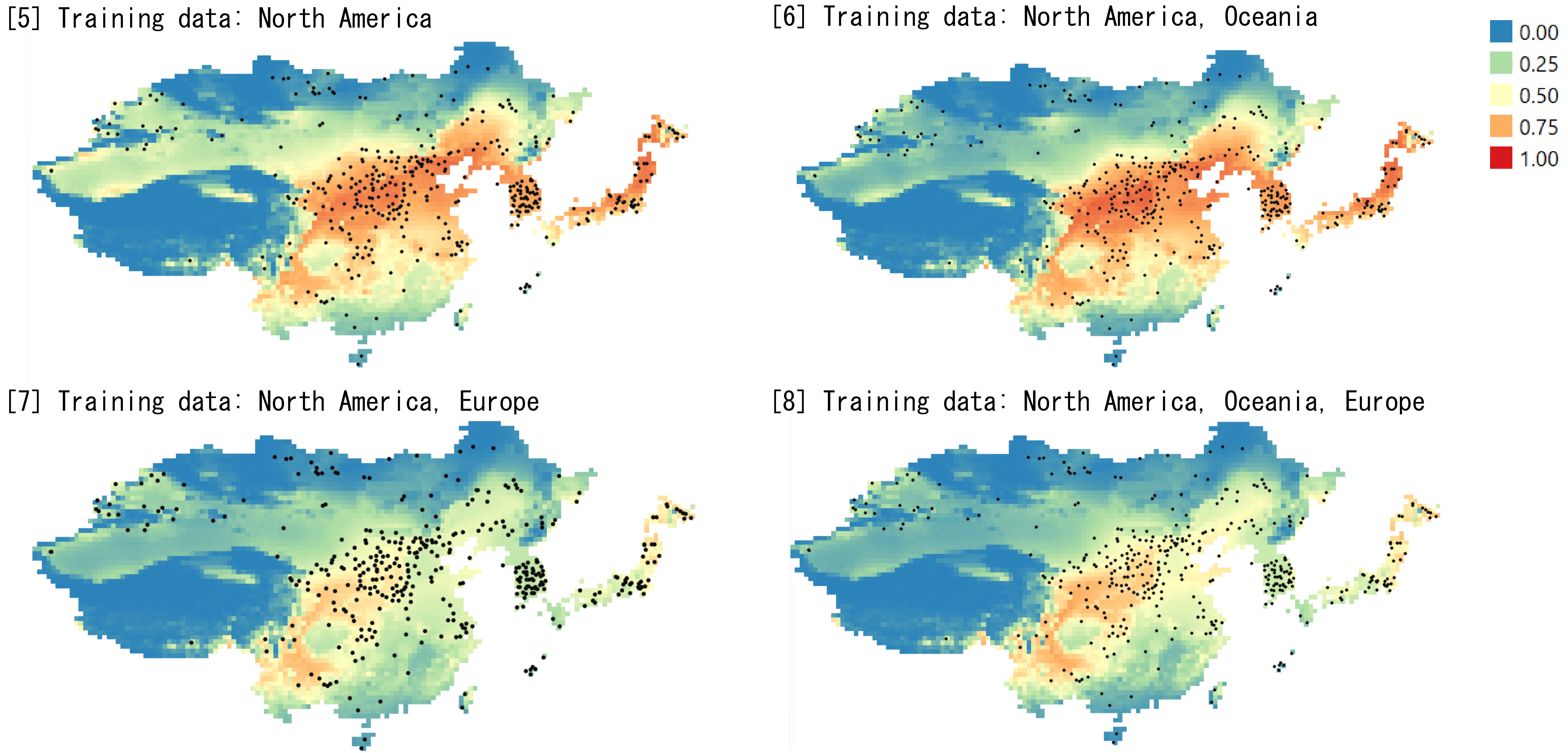

Figure S3. Distribution predictions for *Amaranthus retroflexus* (target region: East Asia)

Maps displaying the Maxent output values; the legend is located in the top right of the figure.

The black dots on the maps represent distribution points. (To make the distribution easier to visualize on the maps, the data were subjected to simple systematic sampling with a reference grid of 30′ resolution.)

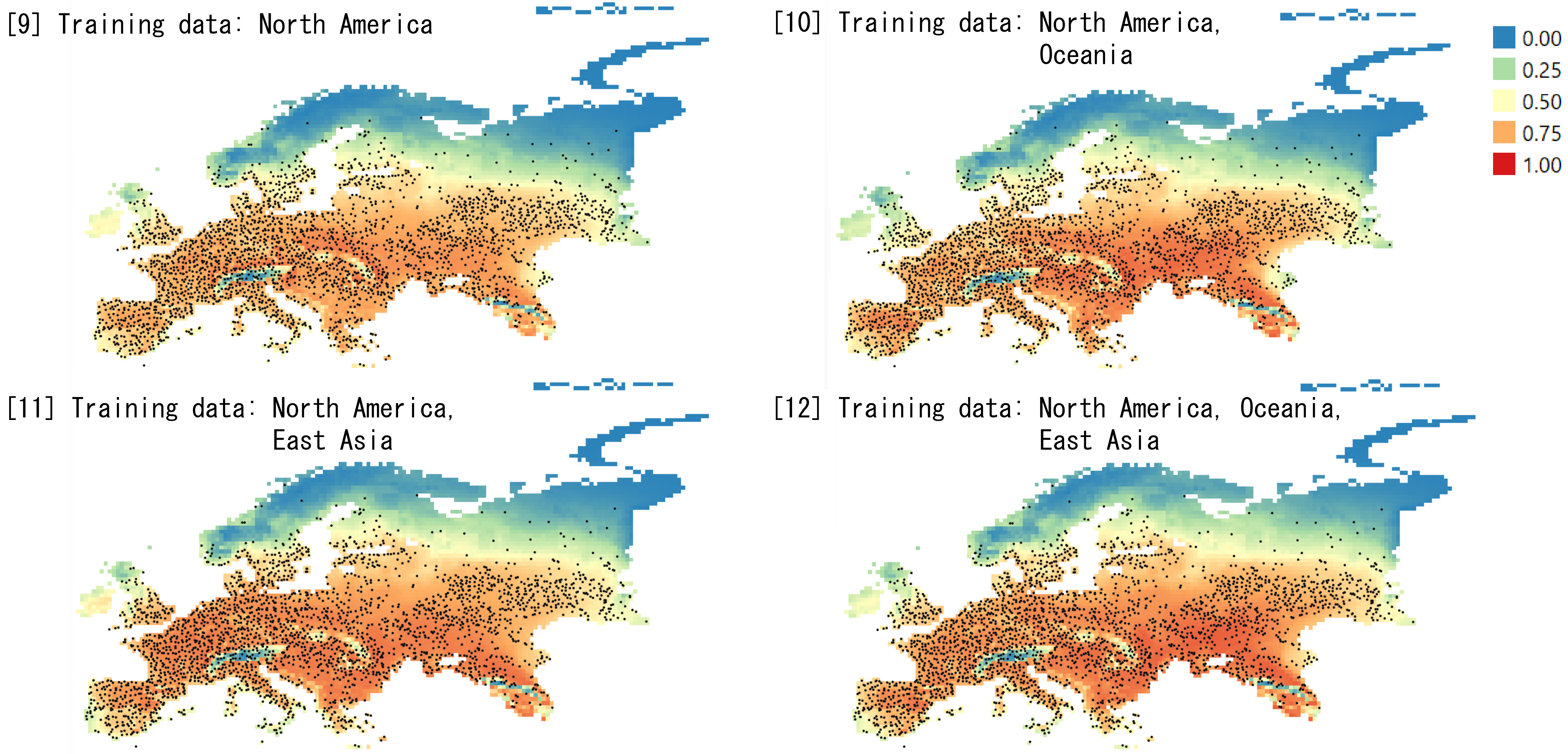

Figure S3. Distribution predictions for *Amaranthus retroflexus* (target region: Europe)

Maps displaying the Maxent output values; the legend is located in the top right of the figure.
The black dots on the maps represent distribution points. (To make the distribution easier to visualize on the maps, the data were subjected to simple systematic sampling with a reference grid of 30′ resolution.)
